# Highly Pathogenic Avian Influenza A(H5N1) Clade 2.3.4.4b Virus in Wild Birds, Chile

**DOI:** 10.3201/eid2909.230067

**Published:** 2023-09

**Authors:** Naomi Ariyama, Catalina Pardo-Roa, Gabriela Muñoz, Carolina Aguayo, Claudia Ávila, Christian Mathieu, Leonardo I. Almonacid, Rafael A. Medina, Barbara Brito, Magdalena Johow, Victor Neira

**Affiliations:** Universidad de Chile, Santiago, Chile (N. Ariyama, G. Muñoz, V. Neira);; Pontificia Universidad Católica de Chile, Santiago (C. Pardo-Roa, L.I. Almonacid, R.A. Medina);; Servicio Agrícola y Ganadero (SAG), Santiago (C. Aguayo, C. Ávila, C. Mathieu, M. Johow);; Emory University, Atlanta, Georgia, USA (R.A. Medina);; Icahn School of Medicine at Mount Sinai, New York, New York, USA (R.A. Medina);; Department of Primary Industries, Menangle, New South Wales, Australia (B. Brito)

**Keywords:** Avian influenza virus, viruses, zoonoses, influenza, respiratory infections, influenza A virus, H5N1 subtype, wild birds, viral dissemination, Chile

## Abstract

In December 2022, highly pathogenic avian influenza A(H5N1) clade 2.3.4.4b virus emerged in Chile. We detected H5N1 virus in 93 samples and obtained 9 whole-genome sequences of strains from wild birds. Phylogenetic analysis suggests multiple viral introductions into South America. Continued surveillance is needed to assess risks to humans and domestic poultry.

Highly pathogenic avian influenza (HPAI) A(H5N1) viruses grouped within hemagglutinin (HA) gene clade 2.3.4.4b are spreading globally and causing high mortality among domestic and wild birds (*1*). In addition, the viruses have spilled over to several nonavian species, including humans (*2*). To contain HPAI outbreaks, poultry exposed to or infected with HPAI viruses have been culled, resulting in disposal of ≈131 million domestic birds globally in 2022 (*3*). Therefore, HPAI viruses pose a threat not only to public health because of zoonotic potential but also to food security.

In late 2021, HPAI H5N1 virus clade 2.3.4.4b, which had spread predominantly in Europe, Asia, and Africa, was detected in wild birds in North America and, shortly after, in domestic poultry (*3*–*5*). In October 2022, this virus reached South America and was officially reported in Colombia; it later was also reported in Peru, Ecuador, and Venezuela (*2*). We describe detection of this virus clade in wild birds in Chile.

## The Study

**Figure 1:**
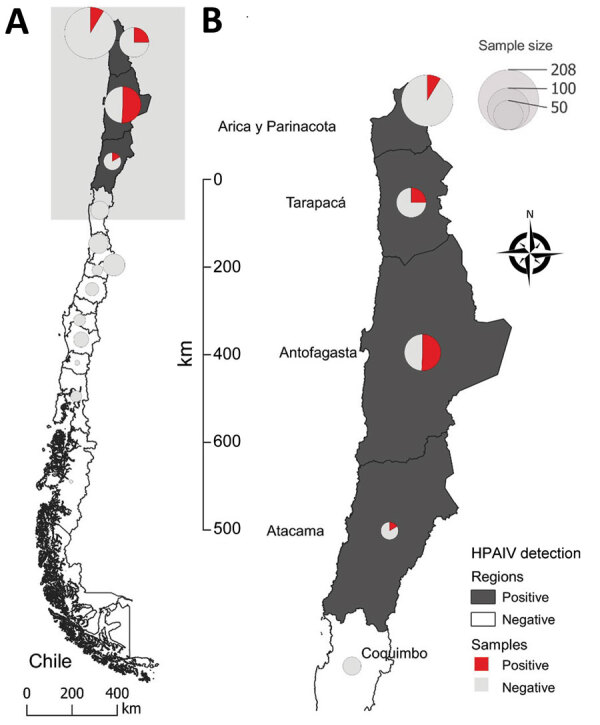
Distribution of samples collected and tested for HPAIV H5N1 clade 2.3.4.4b virus in wild birds, Chile. A) Map of Chile shows regions positive and negative for HPAIV. B) Detail of area in which affected birds were sampled. Size and color of circles indicate sample size and percent positivity. HPAIV, highly pathogenic avian influenza virus.

**Figure 2:**
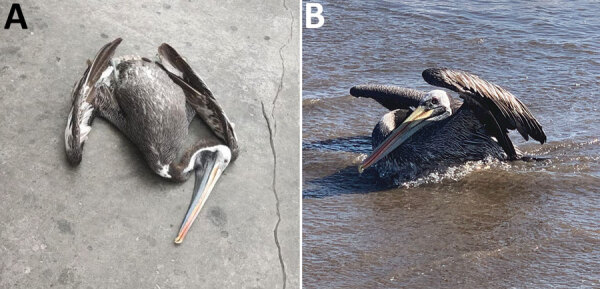
Images of Peruvian pelicans (*Pelecanus thagus*) collected and sampled for highly pathogenic avian influenza virus H5N1 clade 2.3.4.4b, Chile. A) Dead pelican found on land near shoreline; B) ill pelican in water near shoreline.

In early December 2022, increased wild bird deaths were detected across the north coast of Chile (Figure 1). Wild birds, mainly pelicans, were found dead or dying (Figure 2). By December 22, 2022, the official Veterinary Services of the Agricultural and Livestock Service of Chile had collected 1,368 samples for HPAI virus detection and epidemiologic investigation: 1,080 from domestic birds and 288 from wild birds (Appendix Table 1). We performed a total of 578 real-time qualitative reverse transcription PCR (qRT-PCR) reactions to detect active avian influenza virus (AIV) infection and 754 agar gel immunodiffusion (AGID) tests to detect previous AIV exposure (Appendix Table 2).

We initially performed qRT-PCR by using VetMAX-Gold AIV Detection Kit (Applied Biosystems/Thermo Fisher Scientific, https://www.thermofisher.com), targeting the AIV matrix gene. Then, we tested positive samples with specific H5 qRT-PCR according to US Department of Agriculture (USDA) National Veterinary Services Laboratories standard protocols (nos. 1732.02, 1767.01, and 1768.01). We tested 13 tissue samples, 2 (15%) of which were positive; 248 tracheal swab samples, 43 (17%) of which were positive; 314 cloacal swab samples, 47 (15%) of which were positive; and 3 oral swab samples, 1 (33%) of which was positive. Among all samples tested by qRT-PCR, 93 (16%) were H5 AIV–positive with cycle threshold (Ct) values <40. Among positive samples, 18 were from the Arica y Parinacota region, 18 were from Tarapaca, 53 were from Antofagasta, and 4 were from Atacama (Figure 1). No domestic poultry samples were AIV-positive, but among wild bird species, H5 AIV was detected most frequently among Peruvian pelicans (*Pelecanus thagus*) (n = 50, 54%), turkey vultures (*Cathartes aura*) (n = 12, 13%), and Peruvian boobies (*Sula variegata*) (n = 10, 11%) (Appendix Table 1).

We evaluated 754 serum samples by using the official AGID against AIV antigens ribonucleoprotein and matrix protein, according to USDA protocols (https://www.aphis.usda.gov/animal_health/lab_info_services/downloads/Avian_AGID_SOP.pdf) (*6*). We found no positive serum samples (Appendix Table 2). 

We selected 11 H5 AIV–positive samples from the initial outbreak according to Ct values (Ct <27) and location; 9 samples were from pelicans and 2 from gulls, representing 3 administrative regions of Chile. We obtained whole-genome AIV sequences by initial amplification using a multisegment 1-step RT-PCR (*7*), then performed next-generation nanopore sequencing by using the Native Barcoding Kit 96 and MinION platform (Oxford Nanopore Technologies, https://nanoporetech.com), according to the manufacturer’s instructions. We filtered nanopore reads in FASTQ (https://github.com/mcollina/fastq) according to the average quality (Phred score >7) and length <2,600 bp by using NanoFit (*8*). We assembled genomes according to reference by using the nanopore ARTIC pipeline version 1.2.3 (https://artic.network), which we modified by using a relevant reference and to account for the primer sets (Appendix). We used influenza strain A/*Falco*_*rusticolus*/EdoMex/CPA-19638–22/2022(H5N1) (GenBank accession nos. OP691321–28) as the reference. We used the National Center for Biotechnology Information Influenza Virus Sequence Annotation Tool (*9*) to check and annotate assembled genomes and conducted H5 clade classification by using an online subspecies classification tool (*10*). We conducted a BLAST search (https://blast.ncbi.nlm.nih.gov/Blast.cgi) to choose the reference from preliminary assembled contigs constructed with filtered reads that we de novo assembled by using Canu (*11*). We obtained sequences with a mean coverage depth of 33,381× for 10 samples; 9 of 10 genomes were complete (Appendix Table 3).

All positive samples were classified as H5 subtype clade 2.3.4.4b. We inferred Bayesian evolutionary analysis sampling trees for HA and neuraminidase (NA), and maximum-likelihood trees for internal segments (Appendix). A/Peru/LIM-003/2022 and A/Peru/LAM-002/2022 (GISAID accession nos. EPI_ISL_16249730 and EPI_ISL_16249681) were the most closely related HA sequences found in the GISAID database (*12*) (Appendix Figure 1). We observed similar results from phylogenies for NA and internal genes (Appendix Figures 2–8). Sequences from Peru corresponded to isolates collected in November 2022 from domestic chickens from Lima (12′S latitude) and Lambayeque (6′S latitude). On internal gene analyses, HPAI virus sequences available from Ecuador and Mexico grouped closely to the Chile–Peru subcluster (Appendix Figures 3–8). For HA, the Chile–Peru subcluster showed a nonsynonymous mutation, T392A (L131Q), previously associated with antigenic variability in H5N1 virus strains (*13*). We found other synonymous and nonsynonymous mutations in NA (L269M and S339P) and internal genes (Appendix Table 4), but those mutations have not been associated with phenotypic changes.

The phylogenetic tree for the HA segment showed that the sequences from Chile and Peru were closely related to a recent ancestor from North America that was detected during October–November 2022, and the Chile–Peru sequences had closely related ancestors among strains from North America (Appendix Figure 1). A sequence from Ecuador grouped in a paraphyletic branch with different sequences from North America and had a time to most recent common ancestor estimated at August 27, 2022 (95% highest posterior density July 10–September 21). The sequences from Venezuela had a longer branch in the phylogeny reconstruction. Those sequences were more closely related to strains collected earlier in the year from North America and had a time to most recent common ancestor estimated at February 2, 2022 (95% highest posterior density January 15–February 23). The NA, matrix, and polymerase acidic sequences from Venezuela also grouped outside the Chile–Peru subcluster in the maximum-likelihood phylogenies (Appendix Figures 2, 5, 7). The phylogenetic clustering with different sequences from North America suggests that viruses from Venezuela might have resulted from separate introductions into South America. However, because of the low availability of HPAI H5N1 virus sequences from Central and South America, conclusions on the origin of the cluster in Chile are limited. 

Previous studies suggest that the HPAI H5N1 virus clade 2.3.4.4b was introduced into North America multiple times across the East Asia–Australasia/Pacific and Atlantic Flyways and was subsequently disseminated to other flyways (*4*,*14*,*15*). The flyways reach the southernmost tip of South America, representing a high-risk route for HPAIV dissemination across the continent.

## Conclusions

According to official data, as of January 18, 2023, HPAI H5N1 viruses have disseminated as far as the Maule region (35 south latitude) of Chile; no poultry cases have been confirmed. The Agricultural and Livestock Service of Chile implemented a contingency plan to perform extensive surveillance and reinforce biosecurity measurements to avoid introduction of HPAI virus into domestic poultry. The impact of HPAI H5N1 virus in the country, and the potential for introduction of the virus from Chile to Antarctica, remain to be fully elucidated.

This article was preprinted at https://doi.org/10.1101/2023.04.07.535949.

Appendix Additional information on highly pathogenic avian influenza virus H5N1 clade 2.3.4.4b in wild birds, Chile.
