# Supplementary material for: Highly Pathogenic Avian Influenza A(H5N1) Clade 2.3.4.4b Virus in Wild Birds, Chile
Source: Emerg Infect Dis. 2023 Sep;29(9):1842–5. doi: 10.3201/eid2909.230067 (PMC10461661; doi:10.3201/eid2909.230067)
Supplement: Appendix — Additional information on highly pathogenic avian influenza virus H5N1 clade 2.3.4.4b in wild birds, Chile. [file 23-0067-Techapp-s1.pdf]

Article DOI: https://doi.org/10.3201/eid2909.230067

EID cannot ensure accessibility for supplementary materials supplied by authors. Readers who have difficulty accessing supplementary content should contact the authors for assistance.

Highly Pathogenic Avian Influenza H5N1 Clade 2.3.4.4b Virus in Wild Birds, Chile

Appendix

Genome Assembly

Nanopore ARTIC pipeline was changed as follows: first, the reference sequence was changed to the influenza isolate A/*Falco_rusticolus*/EdoMex/CPA-19638–22/2022(H5N1), GenBank accession nos. OP691321 to OP691328. Then, primer bed files were changed to match the PCR primers used to amplify the genome. For the pool field (fifth column) all entries were set to 1. Next, all alignments in the BAM file were used, so option "–normalise" of align_trim software was set to the maximum number of reads. Finally, only medaka consensus with option–RG 1 was used (given that there is only one pool of primers for flu and not two like for SARS-CoV-2); RG 2 was not used.

Phylogenetic Analyses

Datasets for each influenza viral segment sequence were created by retrieving reference sequences from NCBI (*1*) and GISAID databases (*2*). The sequences were aligned using MUSCLE (*3*) in Mega X (*4*). Duplicate sequences were removed, and the phylogeny was estimated using the maximum likelihood method available in IQ-TREE version 2.1.2 (http://www.iqtree.org), employing -m TEST option for nucleotide substitution model selections and 1,000 ultrafast bootstrap replications in CIPRES (*5*,*6*). Each phylogenetic tree was visualized using Figtree (http://tree.bio.ed.ac.uk/software/figtree). Also, we performed Bayesian evolutionary analysis by sampling trees (BEAST) analysis for the HA and NA segment (*7*).

References

1. Johnson M, Zaretskaya I, Raytselis Y, Merezhuk Y, McGinnis S, Madden TL. NCBI BLAST: a better web interface. Nucleic Acids Res. 2008;36(suppl_2):W5-9. PubMed https://doi.org/10.1093/nar/gkn201

2. Khare S, Gurry C, Freitas L, Schultz MB, Bach G, Diallo A, et al. GISAID's Role in Pandemic Response. China CDC Wkly. 2021;3:1049–51. PubMed https://doi.org/10.46234/ccdcw2021.255

3. Edgar RC. MUSCLE: multiple sequence alignment with high accuracy and high throughput. Nucleic Acids Res. 2004;32:1792–7. PubMed https://doi.org/10.1093/nar/gkh340

4. Kumar S, Stecher G, Li M, Knyaz C, Tamura K. MEGA X: Molecular Evolutionary Genetics Analysis across computing platforms. Mol Biol Evol. 2018;35:1547–9. PubMed https://doi.org/10.1093/molbev/msy096

5. Nguyen LT, Schmidt HA, von Haeseler A, Minh BQ. IQ-TREE: a fast and effective stochastic algorithm for estimating maximum-likelihood phylogenies. Mol Biol Evol. 2015;32:268–74. PubMed https://doi.org/10.1093/molbev/msu300

6. Miller MA, Pfeiffer W, Schwartz T. Creating the CIPRES Science Gateway for inference of large phylogenetic trees. Presented at: 2010 Gateway Computing Environments Workshop (GCE); November 14, 2010; New Orleans, Louisiana, USA. https://doi.org/10.1109/GCE.2010.5676129

7. Drummond AJ, Rambaut A. BEAST: Bayesian evolutionary analysis by sampling trees. BMC Evol Biol. 2007;7:214. PubMed https://doi.org/10.1186/1471-2148-7-214

Appendix Table 1. Samples of wild and domestic birds officially analyzed for influenza A virus detection by real time RT-PCR targeting gene M, December 2–22, 2022, Chile*

ID	Collection date	Region	Common name (species)	Category	Sample type	Pooled samples†	RT-PCR	Ct
226518	5 Dec	Metropolitana	ND	Wild or exotic bird	Tracheal swab	1	Negative	NA
226518	5 Dec	Metropolitana	ND	Wild or exotic bird	Tissue	1	Negative	NA
226518	5 Dec	Metropolitana	ND	Wild or exotic bird	Tracheal swab	1	Negative	NA
226518	5 Dec	Metropolitana	ND	Wild or exotic bird	Tissue	1	Negative	NA
226563	2 Dec	Maule	Hawk (*Geranoaetus* sp.)	Wild or exotic bird	Cloacal swab	1	Negative	NA
226615	5 Dec	Tarapaca	Pelican (*Pelecanus thagus*)	Wild or exotic bird	Tracheal swab	1	Positive	26
226615	5 Dec	Tarapaca	Pelican (*P. thagus*)	Wild or exotic bird	Cloacal swab	1	Positive	34
226615	5 Dec	Tarapaca	Pelican (*P. thagus*)	Wild or exotic bird	Tracheal swab	1	Positive	25
226615	5 Dec	Tarapaca	Pelican (*P. thagus*)	Wild or exotic bird	Cloacal swab	1	Positive	36
226618	5 Dec	Arica y Parinacota	Pelican (*P. thagus*)	Wild or exotic bird	Tracheal swab	1	Positive	26.21
226618	5 Dec	Arica y Parinacota	Pelican (*P. thagus*)	Wild or exotic bird	Cloacal swab	1	Positive	26.03
226665	4 Dec	Antofagasta	Pelican (*P. thagus*)	Wild or exotic bird	Cloacal swab	1	Positive	33
226665	4 Dec	Antofagasta	Pelican (*P. thagus*)	Wild or exotic bird	Cloacal swab	1	Positive	32
226666	5 Dec	Antofagasta	Pelican (*P. thagus*)	Wild or exotic bird	Tracheal swab	0	Negative	NA
226666	5 Dec	Antofagasta	Pelican (*P. thagus*)	Wild or exotic bird	Cloacal swab	0	Negative	NA
226666	5 Dec	Antofagasta	Pelican (*P. thagus*)	Wild or exotic bird	Tracheal swab	0	Positive	27
226666	5 Dec	Antofagasta	Pelican (*P. thagus*)	Wild or exotic bird	Cloacal swab	0	Positive	20
226666	5 Dec	Antofagasta	Pelican (*P. thagus*)	Wild or exotic bird	Tracheal swab	1	Negative	NA
226666	5 Dec	Antofagasta	Pelican (*P. thagus*)	Wild or exotic bird	Cloacal swab	1	Negative	NA
226666	5 Dec	Antofagasta	Pelican (*P. thagus*)	Wild or exotic bird	Tracheal swab	1	Positive	25.5
226666	5 Dec	Antofagasta	Pelican (*P. thagus*)	Wild or exotic bird	Cloacal swab	1	Positive	18
226769	5 Dec	Atacama	Northern giant petrel (*Macronectes halli*)	Wild or exotic bird	Oral swab	0	Negative	NA
226769	5 Dec	Atacama	Northern giant petrel (*M. halli*)	Wild or exotic bird	Cloacal swab	0	Negative	NA
226776	6 Dec	Metropolitana	ND	Wild or exotic bird	Tracheal swab	1	Negative	NA
226776	6 Dec	Metropolitana	ND	Wild or exotic bird	Cloacal swab	1	Negative	NA
226776	6 Dec	Metropolitana	ND	Wild or exotic bird	Tissue	1	Negative	NA
226816	6 Dec	Metropolitana	*Hen (G. gallus domesticus)*	Laying hen	Cloacal swab	4	Negative	NA
226816	6 Dec	Metropolitana	*Hen (G. gallus domesticus)*	Laying hen	Cloacal swab	4	Negative	NA
226924	6 Dec	Arica y Parinacota	Pelican (*P. thagus*)	Wild or exotic bird	Tracheal swab	2	Positive	31
226924	6 Dec	Arica y Parinacota	Pelican (*P. thagus*)	Wild or exotic bird	Tracheal swab	0	Positive	33
226924	6 Dec	Arica y Parinacota	Pelican (*P. thagus*)	Wild or exotic bird	Cloacal swab	0	Positive	29
226924	6 Dec	Arica y Parinacota	Pelican (*P. thagus*)	Wild or exotic bird	Cloacal swab	2	Positive	26
226955	6 Dec	Antofagasta	Pelican (*P. thagus*)	Wild or exotic bird	Tracheal swab	1	Positive	18.65

ID	Collection date	Region	Common name (species)	Category	Sample type	Pooled samples†	RT-PCR	Ct
226955	6 Dec	Antofagasta	Pelican (*P. thagus*)	Wild or exotic bird	Cloacal swab	1	Positive	28.97
226955	6 Dec	Antofagasta	Pelican (*P. thagus*)	Wild or exotic bird	Tracheal swab	1	Positive	19.01
226955	6 Dec	Antofagasta	Pelican (*P. thagus*)	Wild or exotic bird	Cloacal swab	1	Positive	33
226958	6 Dec	Antofagasta	Pelican (*P. thagus*)	Wild or exotic bird	Tracheal swab	2	Positive	16.98
226958	6 Dec	Antofagasta	Pelican (*P. thagus*)	Wild or exotic bird	Cloacal swab	2	Positive	25.28
226960	6 Dec	Arica y Parinacota	Franklin's gull (*Leucophaeus pipixcan*)	Wild or exotic bird	Cloacal swab	5	Negative	NA
226960	6 Dec	Arica y Parinacota	Gray gull (*L. modestus*)	Wild or exotic bird	Cloacal swab	5	Negative	NA
226960	6 Dec	Arica y Parinacota	Cinnamon teal (*Anas cyanoptera*)	Wild or exotic bird	Cloacal swab	1	Negative	NA
226960	6 Dec	Arica y Parinacota	Pelican (*P. thagus*)	Wild or exotic bird	Cloacal swab	5	Negative	NA
226963	7 Dec	Maule	American kestrel (*Falco sparverius*)	Wild or exotic bird	Cloacal swab	0	Negative	NA
226963	7 Dec	Maule	American kestrel (*F. sparverius*)	Wild or exotic bird	Tracheal swab	0	Negative	NA
227023	7 Dec	Tarapaca	Pelican (*P. thagus*)	Wild or exotic bird	Cloacal swab	3	Positive	25.38
227023	7 Dec	Tarapaca	Belcher's gull (*Larus belcheri*)	Wild or exotic bird	Cloacal swab	1	Positive	23.63
227023	7 Dec	Tarapaca	Belcher's gull (*L. belcheri*)	Wild or exotic bird	Tracheal swab	1	Positive	21.32
227079	7 Dec	Arica y Parinacota	Peruvean booby (*Sula variegata*)	Wild or exotic bird	Tracheal swab	1	Positive	28
227079	7 Dec	Arica y Parinacota	Peruvean booby (*S. variegata*)	Wild or exotic bird	Cloacal swab	1	Positive	31
227087	7 Dec	Arica y Parinacota	Pelican (*P. thagus*)	Wild or exotic bird	Cloacal swab	2	Positive	25
227087	7 Dec	Arica y Parinacota	Pelican (*P. thagus*)	Wild or exotic bird	Tracheal swab	2	Positive	25
227157	8 Dec	Coquimbo	ND	Wild or exotic bird	Cloacal swab	0	Negative	NA
227198	8 Dec	Arica y Parinacota	Black-crowned night-heron (*Nycticorax nycticorax*)	Wild or exotic bird	Tracheal swab	1	Negative	NA
227198	8 Dec	Arica y Parinacota	Black-crowned night-heron (*N. nycticorax*)	Wild or exotic bird	Tracheal swab	1	Negative	NA
227199	8 Dec	Valparaiso	ND	Wild or exotic bird	Cloacal swab	0	Negative	NA
227199	8 Dec	Valparaiso	ND	Wild or exotic bird	Cloacal swab	0	Negative	NA
227285	9 Dec	Los Lagos	*Hen (G. gallus domesticus)*	Backyard hen	Tracheal swab	3	Negative	NA
227286	9 Dec	Coquimbo	ND	Wild or exotic bird	Cloacal swab	0	Negative	NA
227286	9 Dec	Coquimbo	ND	Wild or exotic bird	Cloacal swab	0	Negative	NA
227286	9 Dec	Coquimbo	ND	Wild or exotic bird	Cloacal swab	0	Negative	NA
227287	9 Dec	Los Lagos	*Hen (G. gallus domesticus)*	Backyard hen	Tracheal swab	3	Negative	NA
227294	9 Dec	Valparaiso	ND	Backyard hen	Cloacal swab	1	Negative	NA
227294	9 Dec	Valparaiso	ND	Backyard hen	Cloacal swab	1	Negative	NA
227294	9 Dec	Valparaiso	ND	Backyard hen	Tracheal swab	1	Negative	NA
227294	9 Dec	Valparaiso	ND	Backyard hen	Tracheal swab	1	Negative	NA
227405	12 Dec	Valparaiso	ND	Wild or exotic bird	Tracheal swab	1	Negative	NA

ID	Collection date	Region	Common name (species)	Category	Sample type	Pooled samples†	RT-PCR	Ct
227405	12 Dec	Valparaiso	ND	Wild or exotic bird	Tracheal swab	1	Negative	NA
227415	9 Dec	Valparaiso	ND	Wild or exotic bird	Cloacal swab	1	Negative	NA
227415	9 Dec	Valparaiso	ND	Wild or exotic bird	Tracheal swab	1	Negative	NA
227423	10 Dec	Antofagasta	Peruvean booby (*S. variegata*)	Wild or exotic bird	Cloacal swab	1	Positive	27.31
227423	10 Dec	Antofagasta	Peruvean booby (*S. variegata*)	Wild or exotic bird	Tracheal swab	1	Positive	27.33
227424	10 Dec	Antofagasta	ND	Wild or exotic bird	Cloacal swab	0	Negative	NA
227424	10 Dec	Antofagasta	Kelp gull (*L. dominicanus*)	Wild or exotic bird	Tracheal swab	1	Negative	NA
227428	10 Dec	Antofagasta	Peruvean booby (*S. variegata*)	Wild or exotic bird	Cloacal swab	1	Positive	31.34
227428	10 Dec	Antofagasta	Peruvean booby (*S. variegata*)	Wild or exotic bird	Tracheal swab	1	Positive	23.39
227435	10 Dec	Antofagasta	Cormorant (*Phalacrocorax* sp.)	Wild or exotic bird	Cloacal swab	1	Negative	NA
227435	10 Dec	Antofagasta	Cormorant (*Phalacrocorax* sp.)	Wild or exotic bird	Tracheal swab	1	Negative	NA
227436	9 Dec	Tarapaca	Pelican (*P. thagus*)	Wild or exotic bird	Tracheal swab	1	Positive	31.56
227436	9 Dec	Tarapaca	Pelican (*P. thagus*)	Wild or exotic bird	Cloacal swab	1	Positive	30.56
227440	9 Dec	Antofagasta	South American tern (*Sterna hirundinacea*)	Wild or exotic bird	Tracheal swab	2	Negative	NA
227442	9 Dec	Tarapaca	ND	Wild or exotic bird	Cloacal swab	5	Positive	35
227480	12 Dec	Bio-Bio	ND	Wild or exotic bird	Tracheal swab	1	Negative	NA
227480	12 Dec	Bio-Bio	ND	Wild or exotic bird	Cloacal swab	1	Negative	NA
227484	11 Dec	Tarapaca	ND	Wild or exotic bird	Cloacal swab	0	Positive	26.78
227484	11 Dec	Tarapaca	ND	Wild or exotic bird	Oral swab	0	Positive	26.29
227487	9 Dec	Metropolitana	ND	Wild or exotic bird	Cloacal swab	1	Negative	NA
227489	11 Dec	Los Lagos	*Hen (G. gallus domesticus)*	Backyard hen	Cloacal swab	2	Negative	NA
227494	10 Dec	Los Lagos	ND	Wild or exotic bird	Tracheal swab	0	Negative	NA
227494	10 Dec	Los Lagos	ND	Wild or exotic bird	Cloacal swab	0	Negative	NA
227512	12 Dec	Metropolitana	ND	Wild or exotic bird	Cloacal swab	1	Negative	NA
227512	12 Dec	Metropolitana	ND	Wild or exotic bird	Cloacal swab	1	Negative	NA
227515	9 Dec	Arica y Parinacota	Gray gull (*L. modestus*)	Wild or exotic bird	Tracheal swab	1	Positive	29.14
227515	9 Dec	Arica y Parinacota	Peruvean booby (*S. variegata*)	Wild or exotic bird	Tracheal swab	1	Positive	26.18
227518	10 Dec	Arica y Parinacota	Harris's hawk (*Parabuteo unicinctus*)	Wild or exotic bird	Cloacal swab	1	Negative	NA
227518	10 Dec	Arica y Parinacota	Harris's hawk (*Parabuteo unicinctus*)	Wild or exotic bird	Tracheal swab	1	Negative	NA
227519	12 Dec	Arica y Parinacota	Inca tern (*Larosterna inca*)	Wild or exotic bird	Cloacal swab	1	Positive	30.97
227519	12 Dec	Arica y Parinacota	Inca tern (*Larosterna inca*)	Wild or exotic bird	Tracheal swab	1	Positive	27.26
227607	12 Dec	Metropolitana	ND	Wild or exotic bird	Cloacal swab	1	Negative	NA
227608	12 Dec	Metropolitana	Yellow-billed pintail (*Anas georgica*)	Wild or exotic bird	Cloacal swab	1	Negative	NA
227631	12 Dec	Metropolitana	Austral thrush (*Turdus falcklandii*)	Wild or exotic bird	Cloacal swab	2	Negative	NA

ID	Collection date	Region	Common name (species)	Category	Sample type	Pooled samples†	RT-PCR	Ct
227631	12 Dec	Metropolitana	Austral thrush (*T. falcklandii*)	Wild or exotic bird	Tracheal swab	2	Negative	NA
227648	13 Dec	Maule	ND	Wild or exotic bird	Cloacal swab	1	Negative	NA
227667	12 Dec	Atacama	Black-faced ibis (*Theristicus melanopis*)	Wild or exotic bird	Tracheal swab	0	Negative	NA
227667	12 Dec	Atacama	Black-faced ibis (*T. melanopis*)	Wild or exotic bird	Cloacal swab	0	Negative	NA
227667	12 Dec	Atacama	Black-faced ibis (*T. melanopis*)	Wild or exotic bird	Tracheal swab	0	Negative	NA
227667	12 Dec	Atacama	Black-faced ibis (*T. melanopis*)	Wild or exotic bird	Cloacal swab	0	Negative	NA
227667	12 Dec	Atacama	Black-faced ibis (*T. melanopis*)	Wild or exotic bird	Tissue	0	Negative	NA
227667	12 Dec	Atacama	Black-faced ibis (*T. melanopis*)	Wild or exotic bird	Tissue	0	Negative	NA
227681	13 Dec	Coquimbo	ND	Wild or exotic bird	Cloacal swab	1	Negative	NA
227729	13 Dec	Antofagasta	Pigeon (*Columba livia*)	Wild or exotic bird	Cloacal swab	0	Negative	NA
227729	13 Dec	Antofagasta	Pigeon (*C. livia*)	Wild or exotic bird	Tracheal swab	1	Negative	NA
227760	13 Dec	Arica y Parinacota	*Hen (G. gallus domesticus)*	Laying hen	Cloacal swab	5	Negative	NA
227760	13 Dec	Arica y Parinacota	*Hen (G. gallus domesticus)*	Laying hen	Cloacal swab	5	Negative	NA
227760	13 Dec	Arica y Parinacota	*Hen (G. gallus domesticus)*	Laying hen	Cloacal swab	5	Negative	NA
227760	13 Dec	Arica y Parinacota	*Hen (G. gallus domesticus)*	Laying hen	Cloacal swab	5	Negative	NA
227760	13 Dec	Arica y Parinacota	*Hen (G. gallus domesticus)*	Laying hen	Cloacal swab	5	Negative	NA
227760	13 Dec	Arica y Parinacota	*Hen (G. gallus domesticus)*	Laying hen	Cloacal swab	5	Negative	NA
227760	13 Dec	Arica y Parinacota	*Hen (G. gallus domesticus)*	Laying hen	Tracheal swab	5	Negative	NA
227760	13 Dec	Arica y Parinacota	*Hen (G. gallus domesticus)*	Laying hen	Tracheal swab	5	Negative	NA
227760	13 Dec	Arica y Parinacota	*Hen (G. gallus domesticus)*	Laying hen	Tracheal swab	5	Negative	NA
227760	13 Dec	Arica y Parinacota	*Hen (G. gallus domesticus)*	Laying hen	Tracheal swab	5	Negative	NA
227760	13 Dec	Arica y Parinacota	*Hen (G. gallus domesticus)*	Laying hen	Tracheal swab	5	Negative	NA
227760	13 Dec	Arica y Parinacota	*Hen (G. gallus domesticus)*	Laying hen	Tracheal swab	5	Negative	NA
227817	14 Dec	Maule	ND	Wild or exotic bird	Cloacal swab	0	Negative	NA
227818	13 Dec	Metropolitana	Raptor (ND)	Wild or exotic bird	Cloacal swab	1	Negative	NA
227826	13 Dec	Atacama	Pelican (*P. thagus*)	Wild or exotic bird	Cloacal swab	0	Positive	31.5
227826	13 Dec	Atacama	Pelican (*P. thagus*)	Wild or exotic bird	Tracheal swab	0	Positive	28
227827	13 Dec	Atacama	Black vulture (*Coragyps atratus*)	Wild or exotic bird	Cloacal swab	0	Negative	NA
227827	13 Dec	Atacama	Black vulture (*C. atratus*)	Wild or exotic bird	Tracheal swab	0	Negative	NA
227837	13 Dec	Tarapaca	ND	Wild or exotic bird	Cloacal swab	1	Positive	36.76
227837	13 Dec	Tarapaca	ND	Wild or exotic bird	Cloacal swab	1	Negative	NA
227840	13 Dec	Tarapaca	ND	Wild or exotic bird	Cloacal swab	1	Negative	NA
227840	13 Dec	Tarapaca	ND	Wild or exotic bird	Tracheal swab	1	Negative	NA

ID	Collection date	Region	Common name (species)	Category	Sample type	Pooled samples†	RT-PCR	Ct
227840	13 Dec	Tarapaca	ND	Wild or exotic bird	Cloacal swab	1	Negative	NA
227840	13 Dec	Tarapaca	ND	Wild or exotic bird	Tracheal swab	1	Negative	NA
227840	13 Dec	Tarapaca	ND	Wild or exotic bird	Cloacal swab	1	Positive	28
227840	13 Dec	Tarapaca	ND	Wild or exotic bird	Tracheal swab	1	Positive	27
227842	13 Dec	Tarapaca	ND	Wild or exotic bird	Cloacal swab	1	Positive	30
227842	13 Dec	Tarapaca	ND	Wild or exotic bird	Tracheal swab	1	Negative	NA
227880	14 Dec	Bio-Bio	ND	Wild or exotic bird	Cloacal swab	1	Negative	NA
227892	14 Dec	Los Rios	Domestic goose (*Anser anser domesticus*)	Backyard goose	Tracheal swab	5	Negative	NA
227898	13 Dec	Atacama	Duck (ND)	Wild or exotic bird	Tracheal swab	0	Negative	NA
227898	13 Dec	Atacama	Duck (ND)	Wild or exotic bird	Cloacal swab	0	Negative	NA
227908	14 Dec	Arica y Parinacota	*Hen (G. gallus domesticus)*	Laying hen	Cloacal swab	5	Negative	NA
227908	14 Dec	Arica y Parinacota	*Hen (G. gallus domesticus)*	Laying hen	Cloacal swab	5	Negative	NA
227908	14 Dec	Arica y Parinacota	*Hen (G. gallus domesticus)*	Laying hen	Cloacal swab	5	Negative	NA
227908	14 Dec	Arica y Parinacota	*Hen (G. gallus domesticus)*	Laying hen	Cloacal swab	5	Negative	NA
227908	14 Dec	Arica y Parinacota	*Hen (G. gallus domesticus)*	Laying hen	Cloacal swab	5	Negative	NA
227908	14 Dec	Arica y Parinacota	*Hen (G. gallus domesticus)*	Laying hen	Cloacal swab	5	Negative	NA
227908	14 Dec	Arica y Parinacota	*Hen (G. gallus domesticus)*	Laying hen	Tracheal swab	5	Negative	NA
227908	14 Dec	Arica y Parinacota	*Hen (G. gallus domesticus)*	Laying hen	Tracheal swab	5	Negative	NA
227908	14 Dec	Arica y Parinacota	*Hen (G. gallus domesticus)*	Laying hen	Tracheal swab	5	Negative	NA
227908	14 Dec	Arica y Parinacota	*Hen (G. gallus domesticus)*	Laying hen	Tracheal swab	5	Negative	NA
227908	14 Dec	Arica y Parinacota	*Hen (G. gallus domesticus)*	Laying hen	Tracheal swab	5	Negative	NA
227908	14 Dec	Arica y Parinacota	*Hen (G. gallus domesticus)*	Laying hen	Tracheal swab	5	Negative	NA
227942	14 Dec	Metropolitana	Eared dove (*Zenaida auriculata*)	Wild or exotic bird	Cloacal swab	0	Negative	NA
227955	14 Dec	Metropolitana	Barn owl (*Tyto alba*)	Wild or exotic bird	Cloacal swab	0	Negative	NA
227955	14 Dec	Metropolitana	Chimango caracara (*Milvago chimango*)	Wild or exotic bird	Cloacal swab	2	Negative	NA
227955	14 Dec	Metropolitana	Austral thrush (*Turdus falcklandii*)	Wild or exotic bird	Cloacal swab	0	Negative	NA
227958	14 Dec	Ñuble	Gull (ND)	Wild or exotic bird	Cloacal swab	0	Negative	NA
227958	14 Dec	Ñuble	Gull (ND)	Wild or exotic bird	Tracheal swab	0	Negative	NA
227968	14 Dec	Metropolitana	American kestrel (*F. sparverius*)	Wild or exotic bird	Cloacal swab	1	Negative	NA
227968	14 Dec	Metropolitana	Eared dove (*Z. auriculata*)	Wild or exotic bird	Cloacal swab	6	Negative	NA
227974	14 Dec	Bio-Bio	ND	Wild or exotic bird	Cloacal swab	1	Negative	NA
227977	14 Dec	Bio-Bio	ND	Wild or exotic bird	Cloacal swab	1	Negative	NA
228023	14 Dec	Coquimbo	ND	Wild or exotic bird	Cloacal swab	4	Negative	NA

ID	Collection date	Region	Common name (species)	Category	Sample type	Pooled samples†	RT-PCR	Ct
228035	15 Dec	Valparaiso	Southern lapwing (*Vanellus chilensis*)	Wild or exotic bird	Tracheal swab	1	Negative	NA
228035	15 Dec	Valparaiso	Southern lapwing (*V. chilensis*)	Wild or exotic bird	Cloacal swab	1	Negative	NA
228050	15 Dec	Valparaiso	*Hen (G. gallus domesticus)*	Backyard hen	Tracheal swab	1	Negative	NA
228050	15 Dec	Valparaiso	*Hen (G. gallus domesticus)*	Backyard hen	Cloacal swab	1	Negative	NA
228050	15 Dec	Valparaiso	*Hen (G. gallus domesticus)*	Backyard hen	Tracheal swab	1	Negative	NA
228050	15 Dec	Valparaiso	*Hen (G. gallus domesticus)*	Backyard hen	Cloacal swab	1	Negative	NA
228083	15 Dec	Maule	Gull (ND)	Wild or exotic bird	Cloacal swab	2	Negative	NA
228083	15 Dec	Maule	Gull (ND)	Wild or exotic bird	Oral swab	2	Negative	NA
228161	15 Dec	Metropolitana	*Hen (G. gallus domesticus)*	Backyard hen	Cloacal swab	4	Negative	NA
228161	15 Dec	Metropolitana	*Hen (G. gallus domesticus)*	Backyard hen	Cloacal swab	4	Negative	NA
228164	16 Dec	Metropolitana	Slender-billed parakeet (*Enicognathus leptorhynchus*)	Wild or exotic bird	Cloacal swab	5	Negative	NA
228164	16 Dec	Metropolitana	Slender-billed parakeet (*E. leptorhynchus*)	Wild or exotic bird	Cloacal swab	5	Negative	NA
228168	16 Dec	Metropolitana	Rosella (*Platycercus* sp)	Wild or exotic bird	Cloacal swab	1	Negative	NA
228170	16 Dec	Metropolitana	Chimango caracara (*M. chimango*)	Wild or exotic bird	Cloacal swab	1	Negative	NA
228181	15 Dec	Arica y Parinacota	*Hen (G. gallus domesticus)*	Backyard hen	Tracheal swab	4	Negative	NA
228181	15 Dec	Arica y Parinacota	*Hen (G. gallus domesticus)*	Backyard hen	Cloacal swab	4	Negative	NA
228181	15 Dec	Arica y Parinacota	Duck (*Anas* sp.)	Commercial duck	Tracheal swab	5	Negative	NA
228181	15 Dec	Arica y Parinacota	Duck (*Anas* sp.)	Commercial duck	Cloacal swab	5	Negative	NA
228184	15 Dec	Arica y Parinacota	*Hen (G. gallus domesticus)*	Backyard hen	Tracheal swab	5	Negative	NA
228184	15 Dec	Arica y Parinacota	*Hen (G. gallus domesticus)*	Backyard hen	Tracheal swab	4	Negative	NA
228184	15 Dec	Arica y Parinacota	*Hen (G. gallus domesticus)*	Backyard hen	Cloacal swab	5	Negative	NA
228184	15 Dec	Arica y Parinacota	*Hen (G. gallus domesticus)*	Backyard hen	Cloacal swab	4	Negative	NA
228184	15 Dec	Arica y Parinacota	Guineafowl (*Numida* sp.)	Backyard hen	Tracheal swab	1	Negative	NA
228184	15 Dec	Arica y Parinacota	Guineafowl (*Numida* sp.)	Backyard hen	Cloacal swab	1	Negative	NA
228198	15 Dec	Coquimbo	ND	Wild or exotic bird	Cloacal swab	1	Negative	NA
228203	15 Dec	Coquimbo	ND	Wild or exotic bird	Cloacal swab	1	Negative	NA
228204	15 Dec	Coquimbo	ND	Wild or exotic bird	Cloacal swab	1	Negative	NA
228205	16 Dec	O'Higgins	Turkey (*Meleagris gallopavo*)	Backyard turkey	Cloacal swab	5	Negative	NA
228205	16 Dec	O'Higgins	Duck (*Anas* sp.)	Breeding duck	Cloacal swab	5	Negative	NA
228208	15 Dec	Metropolitana	ND	Wild or exotic bird	Cloacal swab	1	Negative	NA
228208	15 Dec	Metropolitana	ND	Wild or exotic bird	Cloacal swab	1	Negative	NA
228208	15 Dec	Metropolitana	ND	Wild or exotic bird	Cloacal swab	1	Negative	NA

ID	Collection date	Region	Common name (species)	Category	Sample type	Pooled samples†	RT-PCR	Ct
228209	16 Dec	O'Higgins	Chicken (Gallus gallus domesticus)	Commercial chicken	Tissue	5	Negative	NA
228209	16 Dec	O'Higgins	*Chicken (G. gallus domesticus)*	Commercial chicken	Cloacal swab	5	Negative	NA
228209	16 Dec	O'Higgins	*Chicken (G. gallus domesticus)*	Commercial chicken	Tracheal swab	5	Negative	NA
228223	20 Dec	Los Lagos	ND	Wild or exotic bird	Cloacal swab	1	Negative	NA
228227	14 Dec	Arica y Parinacota	Gray gull (*L. modestus*)	Wild or exotic bird	Tracheal swab	1	Positive	26
228227	14 Dec	Arica y Parinacota	Gray gull *(L. modestus*)	Wild or exotic bird	Tracheal swab	1	Positive	20
228228	15 Dec	Arica y Parinacota	American kestrel (*F. sparverius*)	Wild or exotic bird	Tracheal swab	1	Negative	NA
228228	15 Dec	Arica y Parinacota	American kestrel (*F. sparverius*)	Wild or exotic bird	Cloacal swab	1	Negative	NA
228234	16 Dec	Tarapaca	*Hen (G. gallus domesticus)*	Backyard hen	Cloacal swab	5	Negative	NA
228234	16 Dec	Tarapaca	*Hen (G. gallus domesticus)*	Backyard hen	Cloacal swab	3	Negative	NA
228234	16 Dec	Tarapaca	*Hen (G. gallus domesticus)*	Backyard hen	Tracheal swab	5	Negative	NA
228234	16 Dec	Tarapaca	*Hen (G. gallus domesticus)*	Backyard hen	Tracheal swab	3	Negative	NA
228235	16 Dec	Tarapaca	*Hen (G. gallus domesticus)*	Backyard hen	Cloacal swab	3	Negative	NA
228235	16 Dec	Tarapaca	*Hen (G. gallus domesticus)*	Backyard hen	Tracheal swab	3	Negative	NA
228236	16 Dec	Tarapaca	*Hen (G. gallus domesticus)*	Backyard hen	Tracheal swab	2	Negative	NA
228236	16 Dec	Tarapaca	*Hen (G. gallus domesticus)*	Backyard hen	Cloacal swab	2	Negative	NA
228238	16 Dec	Tarapaca	*Hen (G. gallus domesticus)*	Backyard hen	Cloacal swab	5	Negative	NA
228238	16 Dec	Tarapaca	*Hen (G. gallus domesticus)*	Backyard hen	Cloacal swab	4	Negative	NA
228238	16 Dec	Tarapaca	*Hen (G. gallus domesticus)*	Backyard hen	Tracheal swab	5	Negative	NA
228238	16 Dec	Tarapaca	*Hen (G. gallus domesticus)*	Backyard hen	Tracheal swab	4	Negative	NA
228244	16 Dec	Antofagasta	Pelican (*P. thagus*)	Wild or exotic bird	Tissue	0	Positive	24.31
228244	16 Dec	Antofagasta	Pelican (*P. thagus*)	Wild or exotic bird	Tissue	0	Positive	22
228244	16 Dec	Antofagasta	Pelican (*P. thagus*)	Wild or exotic bird	Tracheal swab	0	Positive	20.09
228244	16 Dec	Antofagasta	Pelican (*P. thagus*)	Wild or exotic bird	Cloacal swab	0	Positive	29.52
228244	16 Dec	Antofagasta	Pelican (*P. thagus*)	Wild or exotic bird	Tracheal swab	0	Positive	20.21
228244	16 Dec	Antofagasta	Pelican (*P. thagus*)	Wild or exotic bird	Cloacal swab	0	Negative	NA
228244	16 Dec	Antofagasta	Pelican (*P. thagus*)	Wild or exotic bird	Tracheal swab	0	Positive	20.25
228244	16 Dec	Antofagasta	Pelican (*P. thagus*)	Wild or exotic bird	Cloacal swab	0	Positive	35
228245	15 Dec	Antofagasta	Vulture (ND)	Wild or exotic bird	Tracheal swab	0	Positive	26.1
228245	15 Dec	Antofagasta	Vulture (ND)	Wild or exotic bird	Cloacal swab	0	Positive	26.8
228245	15 Dec	Antofagasta	Vulture (ND)	Wild or exotic bird	Tracheal swab	3	Negative	NA
228245	15 Dec	Antofagasta	Vulture (ND)	Wild or exotic bird	Cloacal swab	3	Positive	29
228245	15 Dec	Antofagasta	Peruvean booby (*S. variegata*)	Wild or exotic bird	Tracheal swab	0	Positive	20.7
228245	15 Dec	Antofagasta	Peruvean booby (*S. variegata*)	Wild or exotic bird	Cloacal swab	0	Positive	20

ID	Collection date	Region	Common name (species)	Category	Sample type	Pooled samples†	RT-PCR	Ct
228246	15 Dec	Antofagasta	Pelican (*P. thagus*)	Wild or exotic bird	Tracheal swab	1	Positive	21.94
228246	15 Dec	Antofagasta	Pelican (*P. thagus*)	Wild or exotic bird	Cloacal swab	1	Positive	33
228246	15 Dec	Antofagasta	Pelican (*P. thagus*)	Wild or exotic bird	Tracheal swab	0	Positive	16.93
228246	15 Dec	Antofagasta	Pelican (*P. thagus*)	Wild or exotic bird	Cloacal swab	0	Positive	34.37
228247	16 Dec	Antofagasta	*Hen (G. gallus domesticus)*	Backyard hen	Tracheal swab	0	Negative	NA
228247	16 Dec	Antofagasta	*Hen (G. gallus domesticus)*	Backyard hen	Cloacal swab	0	Negative	NA
228247	16 Dec	Antofagasta	*Domestic goose (A. anser domesticus)*	Backyard goose	Tracheal swab	0	Negative	NA
228247	16 Dec	Antofagasta	*Domestic goose (A. anser domesticus)*	Backyard goose	Cloacal swab	0	Negative	NA
228247	16 Dec	Antofagasta	*Domestic goose (A. anser domesticus)*	Backyard goose	Tracheal swab	0	Negative	NA
228247	16 Dec	Antofagasta	*Domestic goose (A. anser domesticus)*	Backyard goose	Cloacal swab	0	Negative	NA
228247	16 Dec	Antofagasta	Duck (*Anas* sp.)	Breeding duck	Tracheal swab	0	Negative	NA
228247	16 Dec	Antofagasta	Duck (*Anas* sp.)	Breeding duck	Cloacal swab	0	Negative	NA
228248	17 Dec	Antofagasta	*Domestic goose (A. anser domesticus)*	Backyard goose	Tracheal swab	0	Negative	NA
228248	17 Dec	Antofagasta	*Domestic goose (A. anser domesticus)*	Backyard goose	Cloacal swab	0	Negative	NA
228248	17 Dec	Antofagasta	*Domestic goose (A. anser domesticus)*	Backyard goose	Tissue	0	Negative	NA
228252	17 Dec	Antofagasta	Turkey vulture (*Cathartes aura*)	Wild or exotic bird	Cloacal swab	1	Positive	23
228252	17 Dec	Antofagasta	Turkey vulture (*C. aura*)	Wild or exotic bird	Tracheal swab	1	Positive	26
228255	17 Dec	Antofagasta	Pelican (*P. thagus*)	Wild or exotic bird	Cloacal swab	1	Positive	35
228255	17 Dec	Antofagasta	Pelican (*P. thagus*)	Wild or exotic bird	Tracheal swab	1	Positive	29
228257	17 Dec	La Araucania	Black-faced ibis (*T. melanopis*)	Wild or exotic bird	Cloacal swab	0	Negative	NA
228257	17 Dec	La Araucania	Black-faced ibis (*T. melanopis*)	Wild or exotic bird	Tracheal swab	0	Negative	NA
228258	14 Dec	La Araucania	Swan (ND)	Wild or exotic bird	Cloacal swab	0	Negative	NA
228258	14 Dec	La Araucania	Swan (ND)	Wild or exotic bird	Tracheal swab	0	Negative	NA
228262	17 Dec	Tarapaca	*Hen (G. gallus domesticus)*	Backyard hen	Cloacal swab	2	Negative	NA
228262	17 Dec	Tarapaca	*Hen (G. gallus domesticus)*	Backyard hen	Tracheal swab	2	Negative	NA
228263	17 Dec	Tarapaca	*Hen (G. gallus domesticus)*	Backyard hen	Cloacal swab	1	Negative	NA
228263	17 Dec	Tarapaca	*Hen (G. gallus domesticus)*	Backyard hen	Tracheal swab	1	Negative	NA
228264	17 Dec	Tarapaca	*Hen (G. gallus domesticus)*	Backyard hen	Cloacal swab	5	Negative	NA
228264	17 Dec	Tarapaca	*Hen (G. gallus domesticus)*	Backyard hen	Cloacal swab	4	Negative	NA
228264	17 Dec	Tarapaca	*Hen (G. gallus domesticus)*	Backyard hen	Tracheal swab	5	Negative	NA
228264	17 Dec	Tarapaca	*Hen (G. gallus domesticus)*	Backyard hen	Tracheal swab	4	Negative	NA
228265	17 Dec	Antofagasta	*Hen (G. gallus domesticus)*	Backyard hen	Tracheal swab	0	Negative	NA
228265	17 Dec	Antofagasta	*Hen (G. gallus domesticus)*	Backyard hen	Cloacal swab	0	Negative	NA
228265	17 Dec	Antofagasta	*Hen (G. gallus domesticus)*	Backyard hen	Tissue	0	Negative	NA

ID	Collection date	Region	Common name (species)	Category	Sample type	Pooled samples†	RT-PCR	Ct
228265	17 Dec	Antofagasta	*Hen (G. gallus domesticus)*	Backyard hen	Tracheal swab	0	Negative	NA
228265	17 Dec	Antofagasta	*Hen (G. gallus domesticus)*	Backyard hen	Cloacal swab	0	Negative	NA
228265	17 Dec	Antofagasta	*Hen (G. gallus domesticus)*	Backyard hen	Tissue	0	Negative	NA
228270	18 Dec	Tarapaca	Duck (*Anas* sp.)	Breeding duck	Tracheal swab	5	Negative	NA
228270	18 Dec	Tarapaca	Duck (*Anas* sp.)	Breeding duck	Tracheal swab	4	Negative	NA
228270	18 Dec	Tarapaca	Duck (*Anas* sp.)	Breeding duck	Cloacal swab	5	Negative	NA
228270	18 Dec	Tarapaca	Duck (*Anas* sp.)	Breeding duck	Cloacal swab	4	Negative	NA
228271	18 Dec	Tarapaca	Rooster (Gallus gallus domesticus)	Backyard hen	Tracheal swab	1	Negative	NA
228271	18 Dec	Tarapaca	*Rooster (G. gallus domesticus)*	Backyard hen	Cloacal swab	1	Negative	NA
228272	18 Dec	Antofagasta	Pelican (*P. thagus*)	Wild or exotic bird	Cloacal swab	1	Positive	25
228272	18 Dec	Antofagasta	Pelican (*P. thagus*)	Wild or exotic bird	Tracheal swab	1	Positive	21
228298	16 Dec	Ñuble	Gull (ND)	Wild or exotic bird	Tracheal swab	0	Negative	NA
228298	16 Dec	Ñuble	Gull (ND)	Wild or exotic bird	Cloacal swab	0	Negative	NA
228298	16 Dec	Ñuble	Gull (ND)	Wild or exotic bird	Tracheal swab	0	Negative	NA
228298	16 Dec	Ñuble	Gull (ND)	Wild or exotic bird	Cloacal swab	0	Negative	NA
228316	17 Dec	Arica y Parinacota	*Hen (G. gallus domesticus)*	Backyard hen	Cloacal swab	5	Negative	NA
228316	17 Dec	Arica y Parinacota	*Hen (G. gallus domesticus)*	Backyard hen	Cloacal swab	4	Negative	NA
228316	17 Dec	Arica y Parinacota	*Hen (G. gallus domesticus)*	Backyard hen	Tracheal swab	5	Negative	NA
228316	17 Dec	Arica y Parinacota	*Hen (G. gallus domesticus)*	Backyard hen	Tracheal swab	4	Negative	NA
228317	16 Dec	Arica y Parinacota	*Chicken (G. gallus domesticus)*	Commercial chicken	Cloacal swab	5	Negative	NA
228317	16 Dec	Arica y Parinacota	*Chicken (G. gallus domesticus)*	Commercial chicken	Cloacal swab	5	Negative	NA
228317	16 Dec	Arica y Parinacota	*Chicken (G. gallus domesticus)*	Commercial chicken	Cloacal swab	5	Negative	NA
228317	16 Dec	Arica y Parinacota	*Chicken (G. gallus domesticus)*	Commercial chicken	Cloacal swab	5	Negative	NA
228317	16 Dec	Arica y Parinacota	*Chicken (G. gallus domesticus)*	Commercial chicken	Cloacal swab	5	Negative	NA
228317	16 Dec	Arica y Parinacota	*Chicken (G. gallus domesticus)*	Commercial chicken	Cloacal swab	5	Negative	NA
228318	16 Dec	Antofagasta	Vulture (ND)	Wild or exotic bird	Tracheal swab	0	Negative	NA
228318	16 Dec	Antofagasta	Vulture (ND)	Wild or exotic bird	Cloacal swab	0	Negative	NA
228318	16 Dec	Antofagasta	Pelican (*P. thagus*)	Wild or exotic bird	Tracheal swab	0	Positive	23
228318	16 Dec	Antofagasta	Pelican (*P. thagus*)	Wild or exotic bird	Cloacal swab	0	Positive	31
228318	16 Dec	Antofagasta	Peruvean booby (*S. variegata*)	Wild or exotic bird	Tracheal swab	0	Positive	31
228318	16 Dec	Antofagasta	Peruvean booby (*S. variegata*)	Wild or exotic bird	Cloacal swab	0	Negative	NA
228324	16 Dec	Arica y Parinacota	*Chicken (G. gallus domesticus)*	Commercial chicken	Tracheal swab	5	Negative	NA
228324	16 Dec	Arica y Parinacota	*Chicken (G. gallus domesticus)*	Commercial chicken	Tracheal swab	5	Negative	NA
228324	16 Dec	Arica y Parinacota	*Chicken (G. gallus domesticus)*	Commercial chicken	Tracheal swab	5	Negative	NA

ID	Collection date	Region	Common name (species)	Category	Sample type	Pooled samples†	RT-PCR	Ct
228324	16 Dec	Arica y Parinacota	*Chicken (G. gallus domesticus)*	Commercial chicken	Tracheal swab	5	Negative	NA
228324	16 Dec	Arica y Parinacota	*Chicken (G. gallus domesticus)*	Commercial chicken	Tracheal swab	5	Negative	NA
228324	16 Dec	Arica y Parinacota	*Chicken (G. gallus domesticus)*	Commercial chicken	Tracheal swab	5	Negative	NA
228325	17 Dec	Arica y Parinacota	*Hen (G. gallus domesticus)*	Backyard hen	Cloacal swab	5	Negative	NA
228325	17 Dec	Arica y Parinacota	*Hen (G. gallus domesticus)*	Backyard hen	Cloacal swab	4	Negative	NA
228325	17 Dec	Arica y Parinacota	*Hen (G. gallus domesticus)*	Backyard hen	Tracheal swab	5	Negative	NA
228325	17 Dec	Arica y Parinacota	*Hen (G. gallus domesticus)*	Backyard hen	Tracheal swab	4	Negative	NA
228328	15 Dec	Los Rios	*Hen (G. gallus domesticus)*	Backyard hen	Tracheal swab	1	Negative	NA
228330	18 Dec	Valparaiso	Monk parakeet (*Myiopsitta monachus*)	Wild or exotic bird	Tracheal swab	1	Negative	NA
228330	18 Dec	Valparaiso	Monk parakeet (*M. monachus*)	Wild or exotic bird	Cloacal swab	1	Negative	NA
228333	16 Dec	Arica y Parinacota	*Chicken (G. gallus domesticus)*	Commercial chicken	Cloacal swab	5	Negative	NA
228333	16 Dec	Arica y Parinacota	*Chicken (G. gallus domesticus)*	Commercial chicken	Cloacal swab	5	Negative	NA
228333	16 Dec	Arica y Parinacota	*Chicken (G. gallus domesticus)*	Commercial chicken	Cloacal swab	5	Negative	NA
228333	16 Dec	Arica y Parinacota	*Chicken (G. gallus domesticus)*	Commercial chicken	Cloacal swab	5	Negative	NA
228333	16 Dec	Arica y Parinacota	*Chicken (G. gallus domesticus)*	Commercial chicken	Cloacal swab	5	Negative	NA
228333	16 Dec	Arica y Parinacota	*Chicken (G. gallus domesticus)*	Commercial chicken	Cloacal swab	5	Negative	NA
228334	16 Dec	Valparaiso	ND	Wild or exotic bird	Tracheal swab	1	Negative	NA
228334	16 Dec	Valparaiso	ND	Wild or exotic bird	Cloacal swab	1	Negative	NA
228335	16 Dec	Arica y Parinacota	*Chicken (G. gallus domesticus)*	Commercial chicken	Tracheal swab	5	Negative	NA
228335	16 Dec	Arica y Parinacota	*Chicken (G. gallus domesticus)*	Commercial chicken	Tracheal swab	5	Negative	NA
228335	16 Dec	Arica y Parinacota	*Chicken (G. gallus domesticus)*	Commercial chicken	Tracheal swab	5	Negative	NA
228335	16 Dec	Arica y Parinacota	*Chicken (G. gallus domesticus)*	Commercial chicken	Tracheal swab	5	Negative	NA
228335	16 Dec	Arica y Parinacota	*Chicken (G. gallus domesticus)*	Commercial chicken	Tracheal swab	5	Negative	NA
228335	16 Dec	Arica y Parinacota	*Chicken (G. gallus domesticus)*	Commercial chicken	Tracheal swab	5	Negative	NA
228336	18 Dec	Coquimbo	ND	Wild or exotic bird	Cloacal swab	1	Negative	NA
228338	17 Dec	Arica y Parinacota	*Chicken (G. gallus domesticus)*	Commercial chicken	Cloacal swab	5	Negative	NA
228338	17 Dec	Arica y Parinacota	*Chicken (G. gallus domesticus)*	Commercial chicken	Cloacal swab	5	Negative	NA
228338	17 Dec	Arica y Parinacota	*Chicken (G. gallus domesticus)*	Commercial chicken	Cloacal swab	5	Negative	NA
228338	17 Dec	Arica y Parinacota	*Chicken (G. gallus domesticus)*	Commercial chicken	Cloacal swab	5	Negative	NA
228338	17 Dec	Arica y Parinacota	*Chicken (G. gallus domesticus)*	Commercial chicken	Cloacal swab	5	Negative	NA
228338	17 Dec	Arica y Parinacota	*Chicken (G. gallus domesticus)*	Commercial chicken	Cloacal swab	5	Negative	NA
228340	18 Dec	Coquimbo	ND	Wild or exotic bird	Cloacal swab	1	Negative	NA
228341	17 Dec	Valparaiso	Kelp gull (*Larus dominicanus*)	Wild or exotic bird	Tracheal swab	1	Negative	NA
228341	17 Dec	Valparaiso	Kelp gull (*L. dominicanus*)	Wild or exotic bird	Cloacal swab	1	Negative	NA

ID	Collection date	Region	Common name (species)	Category	Sample type	Pooled samples†	RT-PCR	Ct
228342	18 Dec	Coquimbo	ND	Wild or exotic bird	Cloacal swab	1	Negative	NA
228342	18 Dec	Coquimbo	ND	Wild or exotic bird	Cloacal swab	1	Negative	NA
228345	17 Dec	Arica y Parinacota	*Chicken (G. gallus domesticus)*	Commercial chicken	Tracheal swab	5	Negative	NA
228345	17 Dec	Arica y Parinacota	*Chicken (G. gallus domesticus)*	Commercial chicken	Tracheal swab	5	Negative	NA
228345	17 Dec	Arica y Parinacota	*Chicken (G. gallus domesticus)*	Commercial chicken	Tracheal swab	5	Negative	NA
228345	17 Dec	Arica y Parinacota	*Chicken (G. gallus domesticus)*	Commercial chicken	Tracheal swab	5	Negative	NA
228345	17 Dec	Arica y Parinacota	*Chicken (G. gallus domesticus)*	Commercial chicken	Tracheal swab	5	Negative	NA
228345	17 Dec	Arica y Parinacota	*Chicken (G. gallus domesticus)*	Commercial chicken	Tracheal swab	5	Negative	NA
228350	17 Dec	Arica y Parinacota	*Chicken (G. gallus domesticus)*	Commercial chicken	Cloacal swab	0	Negative	NA
228350	17 Dec	Arica y Parinacota	*Chicken (G. gallus domesticus)*	Commercial chicken	Cloacal swab	0	Negative	NA
228350	17 Dec	Arica y Parinacota	*Chicken (G. gallus domesticus)*	Commercial chicken	Cloacal swab	0	Negative	NA
228350	17 Dec	Arica y Parinacota	*Chicken (G. gallus domesticus)*	Commercial chicken	Cloacal swab	0	Negative	NA
228350	17 Dec	Arica y Parinacota	*Chicken (G. gallus domesticus)*	Commercial chicken	Cloacal swab	0	Negative	NA
228350	17 Dec	Arica y Parinacota	*Chicken (G. gallus domesticus)*	Commercial chicken	Cloacal swab	0	Negative	NA
228351	17 Dec	Arica y Parinacota	*Chicken (G. gallus domesticus)*	Commercial chicken	Tracheal swab	5	Negative	NA
228351	17 Dec	Arica y Parinacota	*Chicken (G. gallus domesticus)*	Commercial chicken	Tracheal swab	5	Negative	NA
228351	17 Dec	Arica y Parinacota	*Chicken (G. gallus domesticus)*	Commercial chicken	Tracheal swab	5	Negative	NA
228351	17 Dec	Arica y Parinacota	*Chicken (G. gallus domesticus)*	Commercial chicken	Tracheal swab	5	Negative	NA
228351	17 Dec	Arica y Parinacota	*Chicken (G. gallus domesticus)*	Commercial chicken	Tracheal swab	5	Negative	NA
228351	17 Dec	Arica y Parinacota	*Chicken (G. gallus domesticus)*	Commercial chicken	Tracheal swab	5	Negative	NA
228358	16 Dec	Coquimbo	ND	Wild or exotic bird	Cloacal swab	1	Negative	NA
228429	19 Dec	Metropolitana	Austral thrush (*T. falcklandii*)	Wild or exotic bird	Cloacal swab	1	Negative	NA
228440	19 Dec	Tarapaca	Turkey vulture (*C. aura*)	Wild or exotic bird	Tracheal swab	1	Positive	31
228440	19 Dec	Tarapaca	Turkey vulture (*C. aura*)	Wild or exotic bird	Cloacal swab	1	Positive	34
228442	17 Dec	Aysen	ND	Wild or exotic bird	Cloacal swab	0	Negative	NA
228444	19 Dec	Atacama	Pelican (*P. thagus*)	Wild or exotic bird	Cloacal swab	0	Positive	26.5
228444	19 Dec	Atacama	Pelican (*P. thagus*)	Wild or exotic bird	Tracheal swab	0	Positive	34.7
228447	19 Dec	Antofagasta	*Hen (G. gallus domesticus)*	Backyard hen	Cloacal swab	4	Negative	NA
228447	19 Dec	Antofagasta	*Hen (G. gallus domesticus)*	Backyard hen	Tracheal swab	4	Negative	NA
228448	19 Dec	Tarapaca	*Hen (G. gallus domesticus)*	Backyard hen	Tracheal swab	5	Negative	NA
228448	19 Dec	Tarapaca	*Hen (G. gallus domesticus)*	Backyard hen	Tracheal swab	3	Negative	NA
228448	19 Dec	Tarapaca	*Hen (G. gallus domesticus)*	Backyard hen	Cloacal swab	5	Negative	NA
228448	19 Dec	Tarapaca	*Hen (G. gallus domesticus)*	Backyard hen	Cloacal swab	3	Negative	NA
228449	19 Dec	Antofagasta	*Hen (G. gallus domesticus)*	Backyard hen	Cloacal swab	5	Negative	NA

ID	Collection date	Region	Common name (species)	Category	Sample type	Pooled samples†	RT-PCR	Ct
228449	19 Dec	Antofagasta	*Hen (G. gallus domesticus)*	Backyard hen	Tracheal swab	5	Negative	NA
228449	19 Dec	Antofagasta	*Hen (G. gallus domesticus)*	Backyard hen	Cloacal swab	4	Negative	NA
228449	19 Dec	Antofagasta	*Hen (G. gallus domesticus)*	Backyard hen	Tracheal swab	4	Negative	NA
228450	19 Dec	Tarapaca	*Hen (G. gallus domesticus)*	Backyard hen	Tracheal swab	2	Negative	NA
228450	19 Dec	Tarapaca	*Hen (G. gallus domesticus)*	Backyard hen	Cloacal swab	2	Negative	NA
228452	19 Dec	Antofagasta	*Hen (G. gallus domesticus)*	Backyard hen	Cloacal swab	5	Negative	NA
228452	19 Dec	Antofagasta	*Hen (G. gallus domesticus)*	Backyard hen	Tracheal swab	5	Negative	NA
228452	19 Dec	Antofagasta	*Hen (G. gallus domesticus)*	Backyard hen	Cloacal swab	5	Negative	NA
228452	19 Dec	Antofagasta	*Hen (G. gallus domesticus)*	Backyard hen	Tracheal swab	5	Negative	NA
228453	19 Dec	Antofagasta	*Hen (G. gallus domesticus)*	Backyard hen	Cloacal swab	5	Negative	NA
228453	19 Dec	Antofagasta	*Hen (G. gallus domesticus)*	Backyard hen	Tracheal swab	5	Negative	NA
228453	19 Dec	Antofagasta	*Hen (G. gallus domesticus)*	Backyard hen	Cloacal swab	5	Negative	NA
228453	19 Dec	Antofagasta	*Hen (G. gallus domesticus)*	Backyard hen	Tracheal swab	5	Negative	NA
228455	19 Dec	Antofagasta	Gray gull (*L. modestus*)	Wild or exotic bird	Tracheal swab	1	Negative	NA
228455	19 Dec	Antofagasta	Gray gull (*L. modestus*)	Wild or exotic bird	Cloacal swab	1	Negative	NA
228456	19 Dec	Bio-Bio	ND	Wild or exotic bird	Cloacal swab	1	Negative	NA
228456	19 Dec	Bio-Bio	ND	Wild or exotic bird	Tracheal swab	0	Negative	NA
228456	19 Dec	Bio-Bio	Kelp gull (*L. dominicanus*)	Wild or exotic bird	Tissue	0	Negative	NA
228457	19 Dec	Bio-Bio	ND	Wild or exotic bird	Cloacal swab	1	Negative	NA
228457	19 Dec	Bio-Bio	Kelp gull (*L. dominicanus*)	Wild or exotic bird	Tracheal swab	0	Negative	NA
228457	19 Dec	Bio-Bio	Kelp gull (*L. dominicanus*)	Wild or exotic bird	Tissue	0	Negative	NA
228498	19 Dec	Atacama	Pelican (*P. thagus*)	Wild or exotic bird	Cloacal swab	2	Negative	NA
228498	19 Dec	Atacama	Pelican (*P. thagus*	Wild or exotic bird	Tracheal swab	2	Negative	NA
228500	19 Dec	Atacama	Pelican (*P. thagus*	Wild or exotic bird	Cloacal swab	0	Negative	NA
228500	19 Dec	Atacama	Pelican (*P. thagus*	Wild or exotic bird	Tracheal swab	0	Negative	NA
228525	19 Dec	Antofagasta	Belcher's gull (*L. belcheri*)	Wild or exotic bird	Cloacal swab	1	Positive	25
228525	19 Dec	Antofagasta	Belcher's gull (*L. belcheri*)	Wild or exotic bird	Tracheal swab	1	Positive	25
228561	20 Dec	Coquimbo	ND	Wild or exotic bird	Cloacal swab	2	Negative	NA
228565	19 Dec	Atacama	Pigeon (*C. livia*)	Wild or exotic bird	Cloacal swab	2	Negative	NA
228565	19 Dec	Atacama	Pigeon (*C. livia*)	Wild or exotic bird	Tracheal swab	2	Negative	NA
228571	18 Dec	Arica y Parinacota	Wilson's petrel (*Oceanites oceanicus*)	Wild or exotic bird	Cloacal swab	2	Negative	NA
228571	18 Dec	Arica y Parinacota	Wilson's petrel (*O. oceanicus*)	Wild or exotic bird	Tracheal swab	2	Negative	NA
228576	20 Dec	Antofagasta	Turkey vulture (*C. aura*)	Wild or exotic bird	Cloacal swab	1	Positive	30
228576	20 Dec	Antofagasta	Turkey vulture (*C. aura*)	Wild or exotic bird	Cloacal swab	1	Positive	27

ID	Collection date	Region	Common name (species)	Category	Sample type	Pooled samples†	RT-PCR	Ct
228576	20 Dec	Antofagasta	Turkey vulture (*C. aura*)	Wild or exotic bird	Cloacal swab	1	Positive	33
228576	20 Dec	Antofagasta	Turkey vulture (*C. aura*)	Wild or exotic bird	Tracheal swab	1	Positive	28
228576	20 Dec	Antofagasta	Turkey vulture (*C. aura*)	Wild or exotic bird	Tracheal swab	1	Negative	NA
228576	20 Dec	Antofagasta	Turkey vulture (*C. aura*)	Wild or exotic bird	Tracheal swab	1	Negative	NA
228579	19 Dec	Arica y Parinacota	Turkey vulture (*C. aura*)	Wild or exotic bird	Cloacal swab	1	Positive	33
228579	19 Dec	Arica y Parinacota	Turkey vulture (*C. aura*)	Wild or exotic bird	Tracheal swab	1	Positive	27
228641	20 Dec	Tarapaca	*Hen (G. gallus domesticus)*	Backyard hen	Cloacal swab	2	Negative	NA
228641	20 Dec	Tarapaca	*Hen (G. gallus domesticus)*	Backyard hen	Tracheal swab	2	Negative	NA
228642	20 Dec	Tarapaca	*Hen (G. gallus domesticus)*	Backyard hen	Cloacal swab	3	Negative	NA
228642	20 Dec	Tarapaca	*Hen (G. gallus domesticus)*	Backyard hen	Tracheal swab	3	Negative	NA
228685	20 Dec	Arica y Parinacota	*Hen (G. gallus domesticus)*	Laying hen	Cloacal swab	5	Negative	NA
228685	20 Dec	Arica y Parinacota	*Hen (G. gallus domesticus)*	Laying hen	Cloacal swab	5	Negative	NA
228685	20 Dec	Arica y Parinacota	*Hen (G. gallus domesticus)*	Laying hen	Cloacal swab	5	Negative	NA
228685	20 Dec	Arica y Parinacota	*Hen (G. gallus domesticus)*	Laying hen	Cloacal swab	5	Negative	NA
228685	20 Dec	Arica y Parinacota	*Hen (G. gallus domesticus)*	Laying hen	Cloacal swab	5	Negative	NA
228685	20 Dec	Arica y Parinacota	*Hen (G. gallus domesticus)*	Laying hen	Cloacal swab	5	Negative	NA
228685	20 Dec	Arica y Parinacota	*Hen (G. gallus domesticus)*	Laying hen	Tracheal swab	5	Negative	NA
228685	20 Dec	Arica y Parinacota	*Hen (G. gallus domesticus)*	Laying hen	Tracheal swab	5	Negative	NA
228685	20 Dec	Arica y Parinacota	*Hen (G. gallus domesticus)*	Laying hen	Tracheal swab	5	Negative	NA
228685	20 Dec	Arica y Parinacota	*Hen (G. gallus domesticus)*	Laying hen	Tracheal swab	5	Negative	NA
228685	20 Dec	Arica y Parinacota	*Hen (G. gallus domesticus)*	Laying hen	Tracheal swab	5	Negative	NA
228685	20 Dec	Arica y Parinacota	*Hen (G. gallus domesticus)*	Laying hen	Tracheal swab	5	Negative	NA
228687	21 Dec	Arica y Parinacota	*Hen (G. gallus domesticus)*	Laying hen	Cloacal swab	5	Negative	NA
228687	21 Dec	Arica y Parinacota	*Hen (G. gallus domesticus)*	Laying hen	Cloacal swab	5	Negative	NA
228687	21 Dec	Arica y Parinacota	*Hen (G. gallus domesticus)*	Laying hen	Cloacal swab	5	Negative	NA
228687	21 Dec	Arica y Parinacota	*Hen (G. gallus domesticus)*	Laying hen	Cloacal swab	5	Negative	NA
228687	21 Dec	Arica y Parinacota	*Hen (G. gallus domesticus)*	Laying hen	Cloacal swab	5	Negative	NA
228687	21 Dec	Arica y Parinacota	*Hen (G. gallus domesticus)*	Laying hen	Cloacal swab	5	Negative	NA
228687	21 Dec	Arica y Parinacota	*Hen (G. gallus domesticus)*	Laying hen	Tracheal swab	5	Negative	NA
228687	21 Dec	Arica y Parinacota	*Hen (G. gallus domesticus)*	Laying hen	Tracheal swab	5	Negative	NA
228687	21 Dec	Arica y Parinacota	*Hen (G. gallus domesticus)*	Laying hen	Tracheal swab	5	Negative	NA
228687	21 Dec	Arica y Parinacota	*Hen (G. gallus domesticus)*	Laying hen	Tracheal swab	5	Negative	NA
228687	21 Dec	Arica y Parinacota	*Hen (G. gallus domesticus)*	Laying hen	Tracheal swab	5	Negative	NA
228687	21 Dec	Arica y Parinacota	*Hen (G. gallus domesticus)*	Laying hen	Tracheal swab	5	Negative	NA

ID	Collection date	Region	Common name (species)	Category	Sample type	Pooled samples†	RT-PCR	Ct
228688	20 Dec	Maule	Kelp gull (*L. dominicanus*)	Wild or exotic bird	Cloacal swab	5	Negative	NA
228688	20 Dec	Maule	Kelp gull (*L. dominicanus*)	Wild or exotic bird	Tracheal swab	5	Negative	NA
228695	20 Dec	Maule	ND	Wild or exotic bird	Tracheal swab	1	Negative	NA
228695	20 Dec	Maule	ND	Wild or exotic bird	Tracheal swab	1	Negative	NA
228695	20 Dec	Maule	ND	Wild or exotic bird	Tracheal swab	1	Negative	NA
228695	20 Dec	Maule	ND	Wild or exotic bird	Cloacal swab	1	Negative	NA
228695	20 Dec	Maule	ND	Wild or exotic bird	Cloacal swab	1	Negative	NA
228696	20 Dec	Metropolitana	Chimango caracara (*M. chimango*)	Wild or exotic bird	Cloacal swab	5	Negative	NA
228696	20 Dec	Metropolitana	Magellanic horned owl (*Bubo magellanicus*)	Wild or exotic bird	Cloacal swab	1	Negative	NA
228696	20 Dec	Metropolitana	American kestrel (*F. sparverius*)	Wild or exotic bird	Cloacal swab	5	Negative	NA
228696	20 Dec	Metropolitana	Austral pygmy owl (*Glaucidium nanum*)	Wild or exotic bird	Cloacal swab	5	Negative	NA
228705	20 Dec	Antofagasta	Black-crowned night-heron (*N. nycticorax*)	Wild or exotic bird	Cloacal swab	1	Positive	36
228705	20 Dec	Antofagasta	Turkey vulture (*C. aura*)	Wild or exotic bird	Cloacal swab	1	Positive	34
228705	20 Dec	Antofagasta	Black-crowned night-heron (*N. nycticorax*)	Wild or exotic bird	Tracheal swab	1	Positive	36.6
228705	20 Dec	Antofagasta	Turkey vulture (*C. aura*)	Wild or exotic bird	Tracheal swab	1	Positive	28
228720	20 Dec	Atacama	Plover (*Charadrius* sp.)	Wild or exotic bird	Cloacal swab	0	Negative	NA
228720	20 Dec	Atacama	Plover (*Charadrius* sp.)	Wild or exotic bird	Tracheal swab	0	Negative	NA
228726	20 Dec	Ñuble	Gull (ND)	Wild or exotic bird	Tracheal swab	1	Negative	NA
228726	20 Dec	Ñuble	Gull (ND)	Wild or exotic bird	Cloacal swab	1	Negative	NA
228726	20 Dec	Ñuble	Gull (ND)	Wild or exotic bird	Cloacal swab	1	Negative	NA
228765	21 Dec	Valparaiso	ND	Wild or exotic bird	Tracheal swab	0	Negative	NA
228765	21 Dec	Valparaiso	ND	Wild or exotic bird	Cloacal swab	0	Negative	NA
228769	20 Dec	Valparaiso	ND	Wild or exotic bird	Tracheal swab	1	Negative	NA
228769	20 Dec	Valparaiso	ND	Wild or exotic bird	Tracheal swab	1	Negative	NA
228784	21 Dec	Valparaiso	Kelp gull (*L. dominicanus*)	Wild or exotic bird	Tracheal swab	1	Negative	NA
228784	21 Dec	Valparaiso	Kelp gull (*L. dominicanus*)	Wild or exotic bird	Cloacal swab	1	Negative	NA
228804	20 Dec	La Araucania	*Hen (G. gallus domesticus)*	Backyard hen	Cloacal swab	0	Negative	NA
228804	20 Dec	La Araucania	*Hen (G. gallus domesticus)*	Backyard hen	Tracheal swab	0	Negative	NA
228804	20 Dec	La Araucania	*Hen (G. gallus domesticus)*	Backyard hen	Cloacal swab	0	Negative	NA
228804	20 Dec	La Araucania	*Hen (G. gallus domesticus)*	Backyard hen	Tracheal swab	0	Negative	NA
228806	21 Dec	Coquimbo	Pelican (*P. thagus*)	Wild or exotic bird	Cloacal swab	1	Negative	NA
228815	21 Dec	Tarapaca	*Hen (G. gallus domesticus)*	Backyard hen	Cloacal swab	5	Negative	NA
228815	21 Dec	Tarapaca	*Hen (G. gallus domesticus)*	Backyard hen	Cloacal swab	4	Negative	NA
228815	21 Dec	Tarapaca	Duck (*Anas* sp.)	Breeding duck	Cloacal swab	4	Negative	NA

ID	Collection date	Region	Common name (species)	Category	Sample type	Pooled samples†	RT-PCR	Ct
228815	21 Dec	Tarapaca	Duck (*Anas* sp.)	Breeding duck	Cloacal swab	5	Negative	NA
228815	21 Dec	Tarapaca	*Hen (G. gallus domesticus)*	Backyard hen	Tracheal swab	5	Negative	NA
228815	21 Dec	Tarapaca	*Hen (G. gallus domesticus)*	Backyard hen	Tracheal swab	4	Negative	NA
228815	21 Dec	Tarapaca	Duck (*Anas* sp.)	Breeding duck	Tracheal swab	5	Negative	NA
228815	21 Dec	Tarapaca	Duck (*Anas* sp.)	Breeding duck	Tracheal swab	4	Negative	NA
228817	21 Dec	Tarapaca	*Hen (G. gallus domesticus)*	Backyard hen	Cloacal swab	3	Negative	NA
228817	21 Dec	Tarapaca	*Hen (G. gallus domesticus)*	Backyard hen	Tracheal swab	3	Negative	NA
228819	21 Dec	Tarapaca	*Hen (G. gallus domesticus)*	Backyard hen	Cloacal swab	1	Negative	NA
228819	21 Dec	Tarapaca	*Hen (G. gallus domesticus)*	Backyard hen	Tracheal swab	1	Negative	NA
228824	21 Dec	Los Lagos	Chimango caracara (*M. chimango*)	Wild or exotic bird	Cloacal swab	1	Negative	NA
228824	21 Dec	Los Lagos	Black-faced ibis (*T. melanopis*)	Wild or exotic bird	Cloacal swab	0	Negative	NA
228825	21 Dec	O'Higgins	Brown-hooded gull (*Chroicocephalus maculipennis*)	Wild or exotic bird	Cloacal swab	3	Negative	NA
228825	21 Dec	O'Higgins	Franklin's gull (*Leucophaeus pipixcan*)	Wild or exotic bird	Cloacal swab	5	Negative	NA
228827	21 Dec	La Araucania	*Hen (G. gallus domesticus)*	Backyard hen	Cloacal swab	0	Negative	NA
228827	21 Dec	La Araucania	*Hen (G. gallus domesticus)*	Backyard hen	Tracheal swab	0	Negative	NA
228827	21 Dec	La Araucania	*Hen (G. gallus domesticus)*	Backyard hen	Cloacal swab	0	Negative	NA
228827	21 Dec	La Araucania	*Hen (G. gallus domesticus)*	Backyard hen	Tracheal swab	0	Negative	NA
228827	21 Dec	La Araucania	*Hen (G. gallus domesticus)*	Backyard hen	Cloacal swab	0	Negative	NA
228827	21 Dec	La Araucania	*Hen (G. gallus domesticus)*	Backyard hen	Tracheal swab	0	Negative	NA
228832	21 Dec	Valparaiso	Kelp gull (*L. dominicanus*)	Wild or exotic bird	Tracheal swab	1	Negative	NA
228832	21 Dec	Valparaiso	Kelp gull (*L. dominicanus*)	Wild or exotic bird	Cloacal swab	1	Negative	NA
228833	21 Dec	Antofagasta	Pelican (*P. thagus*)	Wild or exotic bird	Cloacal swab	1	Positive	30
228833	21 Dec	Antofagasta	Pelican (*P. thagus*)	Wild or exotic bird	Tracheal swab	1	Positive	21
228834	21 Dec	Metropolitana	Barn owl (*T. alba*)	Wild or exotic bird	Cloacal swab	4	Negative	NA
228835	21 Dec	La Araucania	*Domestic goose (A. anser domesticus)*	Backyard goose	Tracheal swab	2	Negative	NA
228835	21 Dec	La Araucania	*Domestic goose (A. anser domesticus)*	Backyard goose	Cloacal swab	2	Negative	NA
228836	21 Dec	La Araucania	*Hen (G. gallus domesticus)*	Backyard hen	Tracheal swab	0	Negative	NA
228836	21 Dec	La Araucania	*Hen (G. gallus domesticus)*	Backyard hen	Cloacal swab	0	Negative	NA
228852	21 Dec	Arica y Parinacota	*Chicken (G. gallus domesticus)*	Commercial chicken	Cloacal swab	0	Negative	NA
228852	21 Dec	Arica y Parinacota	*Chicken (G. gallus domesticus)*	Commercial chicken	Cloacal swab	0	Negative	NA
228852	21 Dec	Arica y Parinacota	*Chicken (G. gallus domesticus)*	Commercial chicken	Tracheal swab	0	Negative	NA
228852	21 Dec	Arica y Parinacota	*Chicken (G. gallus domesticus)*	Commercial chicken	Tracheal swab	0	Negative	NA
228852	21 Dec	Arica y Parinacota	*Chicken (G. gallus domesticus)*	Commercial chicken	Cloacal swab	0	Negative	NA

ID	Collection date	Region	Common name (species)	Category	Sample type	Pooled samples†	RT-PCR	Ct
228852	21 Dec	Arica y Parinacota	*Chicken (G. gallus domesticus)*	Commercial chicken	Cloacal swab	0	Negative	NA
228852	21 Dec	Arica y Parinacota	*Chicken (G. gallus domesticus)*	Commercial chicken	Tracheal swab	0	Negative	NA
228852	21 Dec	Arica y Parinacota	*Chicken (G. gallus domesticus)*	Commercial chicken	Tracheal swab	0	Negative	NA
228852	21 Dec	Arica y Parinacota	*Chicken (G. gallus domesticus)*	Commercial chicken	Cloacal swab	0	Negative	NA
228852	21 Dec	Arica y Parinacota	*Chicken (G. gallus domesticus)*	Commercial chicken	Cloacal swab	0	Negative	NA
228852	21 Dec	Arica y Parinacota	*Chicken (G. gallus domesticus)*	Commercial chicken	Tracheal swab	0	Negative	NA
228852	21 Dec	Arica y Parinacota	*Chicken (G. gallus domesticus)*	Commercial chicken	Tracheal swab	0	Negative	NA
228854	21 Dec	Arica y Parinacota	*Chicken (G. gallus domesticus)*	Commercial chicken	Cloacal swab	0	Negative	NA
228854	21 Dec	Arica y Parinacota	*Chicken (G. gallus domesticus)*	Commercial chicken	Cloacal swab	0	Negative	NA
228854	21 Dec	Arica y Parinacota	*Chicken (G. gallus domesticus)*	Commercial chicken	Tracheal swab	0	Negative	NA
228854	21 Dec	Arica y Parinacota	*Chicken (G. gallus domesticus)*	Commercial chicken	Tracheal swab	0	Negative	NA
228854	21 Dec	Arica y Parinacota	*Chicken (G. gallus domesticus)*	Commercial chicken	Cloacal swab	0	Negative	NA
228854	21 Dec	Arica y Parinacota	*Chicken (G. gallus domesticus)*	Commercial chicken	Cloacal swab	0	Negative	NA
228854	21 Dec	Arica y Parinacota	*Chicken (G. gallus domesticus)*	Commercial chicken	Tracheal swab	0	Negative	NA
228854	21 Dec	Arica y Parinacota	*Chicken (G. gallus domesticus)*	Commercial chicken	Tracheal swab	0	Negative	NA
228854	21 Dec	Arica y Parinacota	*Chicken (G. gallus domesticus)*	Commercial chicken	Cloacal swab	0	Negative	NA
228854	21 Dec	Arica y Parinacota	*Chicken (G. gallus domesticus)*	Commercial chicken	Cloacal swab	0	Negative	NA
228854	21 Dec	Arica y Parinacota	*Chicken (G. gallus domesticus)*	Commercial chicken	Tracheal swab	0	Negative	NA
228854	21 Dec	Arica y Parinacota	*Chicken (G. gallus domesticus)*	Commercial chicken	Tracheal swab	0	Negative	NA
228858	21 Dec	Arica y Parinacota	*Hen (G. gallus domesticus)*	Backyard hen	Tracheal swab	5	Negative	NA
228858	21 Dec	Arica y Parinacota	*Hen (G. gallus domesticus)*	Backyard hen	Tracheal swab	4	Negative	NA
228858	21 Dec	Arica y Parinacota	*Hen (G. gallus domesticus)*	Backyard hen	Cloacal swab	5	Negative	NA
228858	21 Dec	Arica y Parinacota	*Hen (G. gallus domesticus)*	Backyard hen	Cloacal swab	4	Negative	NA
228860	21 Dec	Arica y Parinacota	*Hen (G. gallus domesticus)*	Backyard hen	Tracheal swab	5	Negative	NA
228860	21 Dec	Arica y Parinacota	*Hen (G. gallus domesticus)*	Backyard hen	Tracheal swab	4	Negative	NA
228860	21 Dec	Arica y Parinacota	*Hen (G. gallus domesticus)*	Backyard hen	Cloacal swab	5	Negative	NA
228860	21 Dec	Arica y Parinacota	*Hen (G. gallus domesticus)*	Backyard hen	Cloacal swab	4	Negative	NA
228862	21 Dec	Arica y Parinacota	*Hen (G. gallus domesticus)*	Backyard hen	Tracheal swab	5	Negative	NA
228862	21 Dec	Arica y Parinacota	*Hen (G. gallus domesticus)*	Backyard hen	Tracheal swab	4	Negative	NA
228862	21 Dec	Arica y Parinacota	*Hen (G. gallus domesticus)*	Backyard hen	Cloacal swab	5	Negative	NA
228862	21 Dec	Arica y Parinacota	*Hen (G. gallus domesticus)*	Backyard hen	Cloacal swab	4	Negative	NA
228868	20 Dec	Los Lagos	ND	Wild or exotic bird	Cloacal swab	0	Negative	NA
228868	20 Dec	Los Lagos	ND	Wild or exotic bird	Tracheal swab	0	Negative	NA
228906	22 Dec	Coquimbo	*Chicken (G. gallus domesticus)*	Commercial chicken	Cloacal swab	2	Negative	NA

ID	Collection date	Region	Common name (species)	Category	Sample type	Pooled samples†	RT-PCR	Ct
228906	22 Dec	Coquimbo	*Chicken (G. gallus domesticus)*	Commercial chicken	Tracheal swab	2	Negative	NA
228911	20 Dec	Coquimbo	Gull (ND)	Wild or exotic bird	Cloacal swab	1	Negative	NA
228919	20 Dec	Coquimbo	Gull (ND)	Wild or exotic bird	Cloacal swab	1	Negative	NA
228933	21 Dec	Ñuble	Gull (ND)	Wild or exotic bird	Tracheal swab	1	Negative	NA
228933	21 Dec	Ñuble	Gull (ND)	Wild or exotic bird	Cloacal swab	1	Negative	NA
228938	21 Dec	Coquimbo	Gull (ND)	Wild or exotic bird	Cloacal swab	1	Negative	NA
228949	21 Dec	Coquimbo	Gull (ND)	Wild or exotic bird	Cloacal swab	1	Negative	NA
228963	21 Dec	Coquimbo	Gull (ND)	Wild or exotic bird	Cloacal swab	1	Negative	NA
228969	22 Dec	Arica y Parinacota	*Chicken (G. gallus domesticus)*	Commercial chicken	Cloacal swab	0	Negative	NA
228969	22 Dec	Arica y Parinacota	*Chicken (G. gallus domesticus)*	Commercial chicken	Cloacal swab	0	Negative	NA
228969	22 Dec	Arica y Parinacota	*Chicken (G. gallus domesticus)*	Commercial chicken	Tracheal swab	0	Negative	NA
228969	22 Dec	Arica y Parinacota	*Chicken (G. gallus domesticus)*	Commercial chicken	Tracheal swab	0	Negative	NA
228969	22 Dec	Arica y Parinacota	*Chicken (G. gallus domesticus)*	Commercial chicken	Cloacal swab	0	Negative	NA
228969	22 Dec	Arica y Parinacota	*Chicken (G. gallus domesticus)*	Commercial chicken	Cloacal swab	0	Negative	NA
228969	22 Dec	Arica y Parinacota	*Chicken (G. gallus domesticus)*	Commercial chicken	Tracheal swab	0	Negative	NA
228969	22 Dec	Arica y Parinacota	*Chicken (G. gallus domesticus)*	Commercial chicken	Tracheal swab	0	Negative	NA
228972	21 Dec	Coquimbo	Gull (ND)	Wild or exotic bird	Cloacal swab	1	Negative	NA
228974	22 Dec	Arica y Parinacota	*Chicken (G. gallus domesticus)*	Commercial chicken	Cloacal swab	0	Negative	NA
228974	22 Dec	Arica y Parinacota	*Chicken (G. gallus domesticus)*	Commercial chicken	Cloacal swab	0	Negative	NA
228974	22 Dec	Arica y Parinacota	*Chicken (G. gallus domesticus)*	Commercial chicken	Tracheal swab	0	Negative	NA
228974	22 Dec	Arica y Parinacota	*Chicken (G. gallus domesticus)*	Commercial chicken	Tracheal swab	0	Negative	NA
228974	22 Dec	Arica y Parinacota	*Chicken (G. gallus domesticus)*	Commercial chicken	Cloacal swab	0	Negative	NA
228974	22 Dec	Arica y Parinacota	*Chicken (G. gallus domesticus)*	Commercial chicken	Cloacal swab	0	Negative	NA
228974	22 Dec	Arica y Parinacota	*Chicken (G. gallus domesticus)*	Commercial chicken	Tracheal swab	0	Negative	NA
228974	22 Dec	Arica y Parinacota	*Chicken (G. gallus domesticus)*	Commercial chicken	Tracheal swab	0	Negative	NA
228974	22 Dec	Arica y Parinacota	*Chicken (G. gallus domesticus)*	Commercial chicken	Cloacal swab	0	Negative	NA
228974	22 Dec	Arica y Parinacota	*Chicken (G. gallus domesticus)*	Commercial chicken	Tracheal swab	0	Negative	NA
228974	22 Dec	Arica y Parinacota	*Chicken (G. gallus domesticus)*	Commercial chicken	Tracheal swab	0	Negative	NA
228974	22 Dec	Arica y Parinacota	*Chicken (G. gallus domesticus)*	Commercial chicken	Cloacal swab	0	Negative	NA
228978	21 Dec	Coquimbo	Gull (ND)	Wild or exotic bird	Cloacal swab	2	Negative	NA
228981	21 Dec	Coquimbo	Gull (ND)	Wild or exotic bird	Cloacal swab	1	Negative	NA
229009	22 Dec	O’Higgins	ND	Wild or exotic bird	Tracheal swab	3	Negative	NA
229009	22 Dec	O’Higgins	ND	Wild or exotic bird	Cloacal swab	3	Negative	NA
229098	22 Dec	Arica y Parinacota	*Hen (G. gallus domesticus)*	Backyard hen	Tracheal swab	5	Negative	NA

ID	Collection date	Region	Common name (species)	Category	Sample type	Pooled samples†	RT-PCR	Ct
229098	22 Dec	Arica y Parinacota	*Hen (G. gallus domesticus)*	Backyard hen	Tracheal swab	4	Negative	NA
229098	22 Dec	Arica y Parinacota	*Hen (G. gallus domesticus)*	Backyard hen	Cloacal swab	5	Negative	NA
229098	22 Dec	Arica y Parinacota	*Hen (G. gallus domesticus)*	Backyard hen	Cloacal swab	4	Negative	NA
229100	22 Dec	Arica y Parinacota	*Hen (G. gallus domesticus)*	Backyard hen	Tracheal swab	5	Negative	NA
229100	22 Dec	Arica y Parinacota	*Hen (G. gallus domesticus)*	Backyard hen	Tracheal swab	4	Negative	NA
229100	22 Dec	Arica y Parinacota	*Hen (G. gallus domesticus)*	Backyard hen	Cloacal swab	5	Negative	NA
229100	22 Dec	Arica y Parinacota	*Hen (G. gallus domesticus)*	Backyard hen	Cloacal swab	4	Negative	NA

*Ct, cycle threshold; NA, not applicable; ND, not determined; RT-PCR, reverse transcription PCR.
†Pooled samples correspond to paired cloacal and tracheal swabs for wild bird surveillance; for commercial birds, first detection could be performed using pooled samples of up to 5 tracheal or cloacal swabs per location.

Appendix Table 2. Number of serum samples of domestic and wild birds tested with agar gel immunodiffusion tests (AGID) against Influenza A virus antigens, December 2022, Chile*

Species	No. serum samples
Domestic birds, total	749
Laying hen (*Gallus gallus domesticus*)	542
Backyard hen (*G. gallus domesticus*)	200
Backyard goose (*Anser anser domesticus*)	4
Domestic duck (*Anas platyrhynchos domesticus*)	3
Wild birds, total	5
Gull (*Larus* sp.)	3
American kestrel (*Falco sparverius*)	1
Species not determined	1
Total	754

*Samples tested with agar gel immunodiffusion tests against Influenza A virus antigens (ribonucleoprotein and matrix protein). No positive samples were found from December 1–22, 2022.

Appendix Table 3. Summary of sequencing results of highly pathogenic avian influenza virus H5N1 clade 2.3.4.4b in wild birds, December 2022, Chile*

Strain name, segment	Region	Sample type	Date collected	Accession no.	% Identity	Coverage
A/Pelican/Chile/226618–1/2022 (H5N1)	Arica y Parinacota	Tracheal swab	5 Dec			
PB2				WDE94882	100	11,924x
PB1				WDE94883	100	3,013x
PA				WDE94909	99.73	8,341x
HA				WDE94885	100	9,650x
NP				WDE94886	100	11,808x
NA				WDE94887	100	4,151x
MP				WDE94911	99.90	38,367x
NS				WDE94888	100	28,167x
A/Pelican/Chile/226618–2/2022 (H5N1)	Arica y Parinacota	Cloacal swab	5 Dec			
PB2				WDE94927	99.91	19,866x
PB1				WDE94928	99.82	8,897x
PA				WDE94930	99.91	10,177x
HA				WDE94876	100	15,014x
NP				WDE94932	99.87	28,201x
NA				WDE94877	100	8,202x
MP				WDE94878	100	64,183x
NS				WDE94880	100	44,695x
A/Pelican/Chile/226924–1/2022 (H5N1)	Arica y Parinacota	Tracheal swab	6 Dec			
PB2				WDE94956	99.91	26,631x

Strain name, segment	Region	Sample type	Date collected	Accession no.	% Identity	Coverage
PB1				WDE94957	99.91	41,339x
PA				WDE94959	99.82	7,299x
HA				WDE94961	99.77	23,213x
NP				WDE94962	99.87	33,517x
NA				WDE94899	100	26,486x
MP				WDE94900	100	131,210x
NS				WDE94902	100	231,516x
A/Pelican/Chile/226955–1/2022 (H5N1)	Antofagasta	Tracheal swab	6 Dec			
PB2				WDE94854	100	8,581x
PB1				WDG26816	94.83	5,267x
PA				WDG26818	99.82	21,918x
HA				WDE94855	100	5,363x
NP				WDE94856	100	2,078x
NA				WDE94857	100	1,258x
MP				WDE94858	100	5,045x
NS				WDE94860	100	19,452x
A/Pelican/Chile/226955–3/2022 (H5N1)	Antofagasta	Tracheal swab	6 Dec			
PB2				WDE94933	100	15,234x
PB1				WDE94934	100	2,400x
PA				WDE94936	100	5,342x
HA				WDE94862	99.94	6,789x
NP				WDE94938	100	3,245x
NA				WDE94939	100	1,764x
MP				WDE94940	100	13,239x
NS				WDE94942	100	29,841x
A/Pelican/Chile/226958–1/2022 (H5N1)	Antofagasta	Tracheal swab	6 Dec			
PB2				WDE94890	100	16,410x
PB1				WDE94891	100	7,405x
PA				WDE94868	99.82	12,479x
HA				WDE94893	100	32,988x
NP				WDE94894	100	21,974x
NA				WDE94870	99.93	3,629x
MP				WDE94895	100	47,435x
NS				WDE94897	100	88,402x
A/Pelican/Chile/227023–1/2022 (H5N1)	Tarapaca	Cloacal swab	7 Dec			
PB2				WDG26820	99.91	21x
PB1				WDG26821	54.80	11x
PA				WDG26822	67.30	12x
HA				WDG26823	99.88	21x
NP				WDE94871	100	36x
NA				None	0	6x
MP				WDE94872	100	62x
NS				WDE94874	100	71x
A/Gull/Chile/227023–2/2022 (H5N1)	Tarapaca	Cloacal swab	7 Dec			
PB2				WDE94913	99.82	74,512x
PB1				WDE94914	99.82	29,885x
PA				WDE94916	99.73	37,738x
HA				WDE94918	99.94	44,233x
NP				WDE94919	99.87	97,753x
NA				WDE94904	100	21,346x
MP				WDE94905	100	166,400x
NS				WDE94907	100	110,761x
A/Gull/Chile/227023–3/2022 (H5N1)	Tarapaca	Tracheal swab	7 Dec			
PB2				WDE94920	99.82	115,591x
PB1				WDE94921	99.65	69,821x
PA				WDE94923	99.82	50,493x
HA				WDE94925	99.82	122,820x
NP				WDE94926	99.74	208,545x
NA				WDE94863	100	70,884x
MP				WDE94864	100	447,943x
NS				WDE94866	100	469,081x
A/Pelican/Chile/227087–1/2022 (H5N1)	Arica y Parinacota	Tracheal swab	7 Dec			
PB2				WDE94944	100	232x

Strain name, segment	Region	Sample type	Date collected	Accession no.	% Identity	Coverage
PB1				WDE94945	100	322x
PA				WDE94947	100	388x
HA				WDE94949	100	726x
NP				WDE94950	100	1,213x
NA				WDE94951	100	562x
MP				WDE94952	100	4,441x
NS				WDE94954	100	6,880x

*Species correspond to Peruvian pelican (*Pelecanus thagus)* and Belcher's gull (*Larus belcheri)*. HA, hemagglutinin; MP, matrix protein; NA, neuraminidase; NP, nucleoprotein; NS, nonstructural; PA, polymerase acidic; PB1, polymerase basic protein 1; PB2, polymerase basic protein 2.

Appendix Table 4. Featured mutations found in highly pathogenic avian influenza H5N1 viral strains from Chile sequenced in this study compared with all stains included in the dataset of each viral segment phylogenetic analyses*

Segment	Nucleotide	Synonymous	Amino acid	Comments
HA	T392A	No	L131Q	Unique for the cluster containing Chilean strains
	C549T	Yes		Unique for the cluster containing Chilean strains
	G1686A	Yes		Unique for the cluster containing Chilean strains
NA	T805A	No	L269M	Unique for the cluster containing Chilean strains
	T1015C	No	S339P	Unique for the cluster containing Chilean strains
	C444A	Yes		Unique for the cluster containing Chilean strains
	G696T	Yes		Unique for the cluster containing Chilean strains
M	A254G	No	N85S	Unique for the cluster containing Chilean strains
	A260C	No	N87T	Unique for Peruvian and Chilean strains
	G333T	Yes		Unique for Peruvian and Chilean strains
PB2	A1846G	No	I616V	Unique for the cluster containing Chilean strains
	C2035T	No	P679S	Unique for A/Pelican/Chile/226955–1/2022(H5N1)
	A1155T	Yes		Unique for Chilean strains
	A1846G	Yes		Unique for the cluster containing Chilean strains
PB1	C1132A	No	L378M	Unique for Peruvian and Chilean strains
	G1196A	No	G399D	Unique for Peruvian and Chilean strains
	T1542C	No	S515A	Unique for Chilean strains except A/Pelican/Chile/226924–1/2022(H5N1) and A/Seagull/Chile/227023–3/2022(H5N1)
PA	G170A	No	R53Q	Unique for South American strains
NS1	T346A	No	C116S	Unique for Peruvian and Chilean strains
	C668A	No	A223E	Unique for the cluster containing Chilean strains
	G721T	No	A241S	Unique for Peruvian and Chilean strains
NP	T688C	No	F230L	Unique for Peruvian and Chilean strains
	C1317T	Yes		Unique for Peruvian and Chilean strains

*NCBI (https://www.ncbi.nlm.nih.gov) and GISAID (https://www.gisaid.org) databases. HA, hemagglutinin; M, matrix; NA, neuraminidase; NP, nucleoprotein; NS1, nonstructural protein 1; PA, polymerase acidic; PB1, polymerase basic protein 1; PB2, polymerase basic protein 2

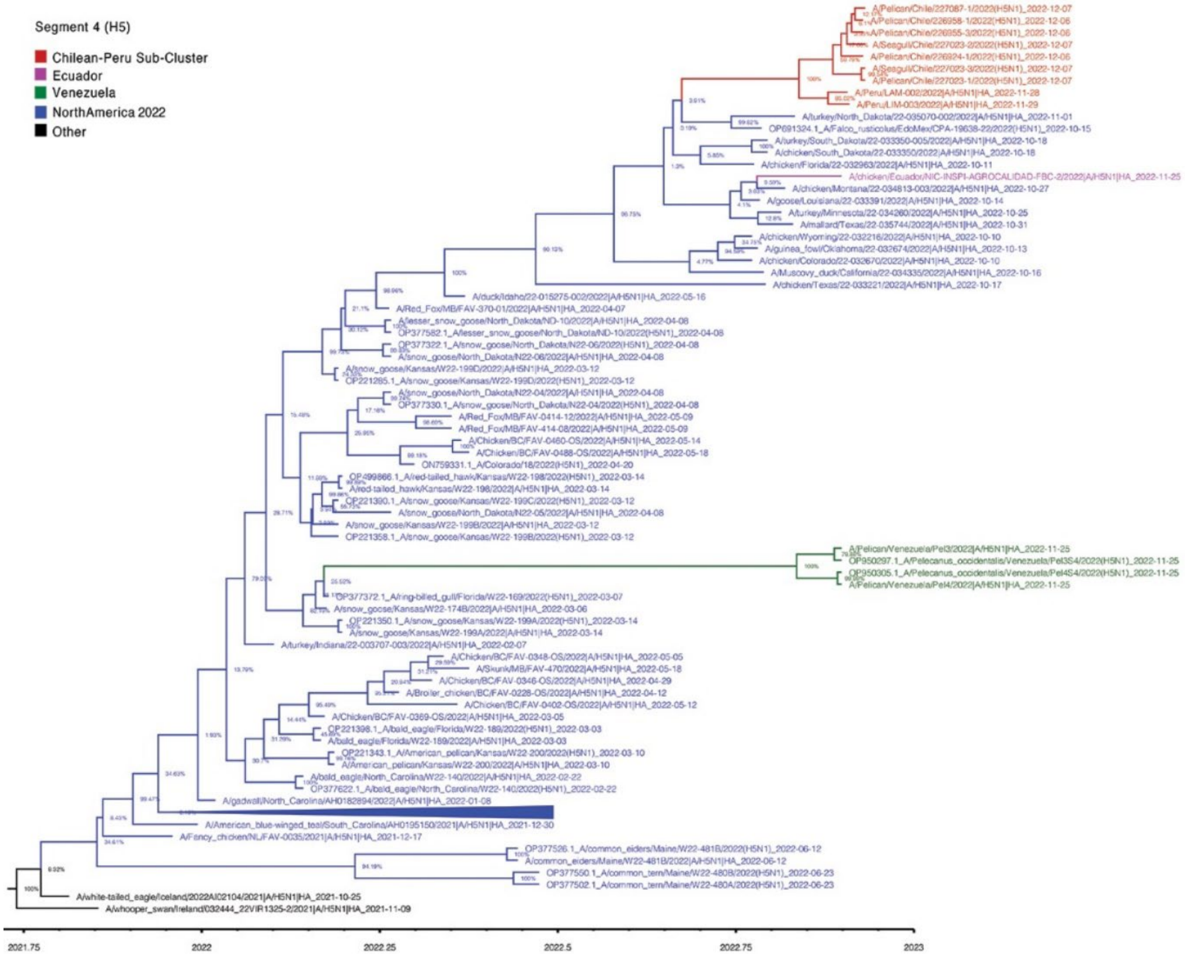

Appendix Figure 1. Bayesian evolutionary analysis sampling tree of hemagglutinin protein of highly pathogenic avian influenza H5N1 subtype 2.3.4.4b clade virus, Chile, 2022. Scale bar indicates nucleotide substitutions per site.

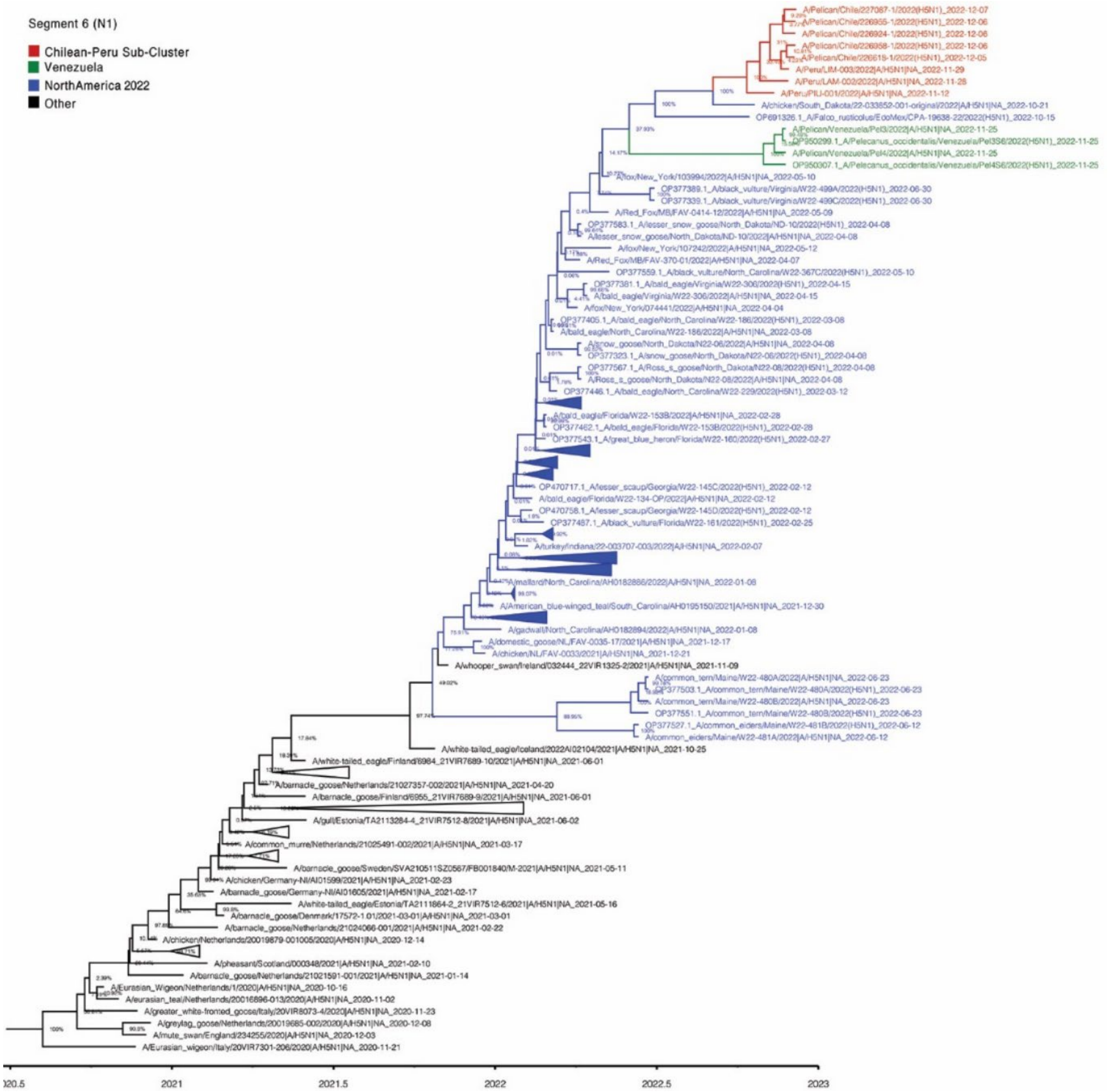

Appendix Figure 2. Bayesian evolutionary analysis sampling tree of neuraminidase protein of highly pathogenic avian influenza H5N1 subtype 2.3.4.4b clade virus, Chile, 2022. Scale bar indicates nucleotide substitutions per site.

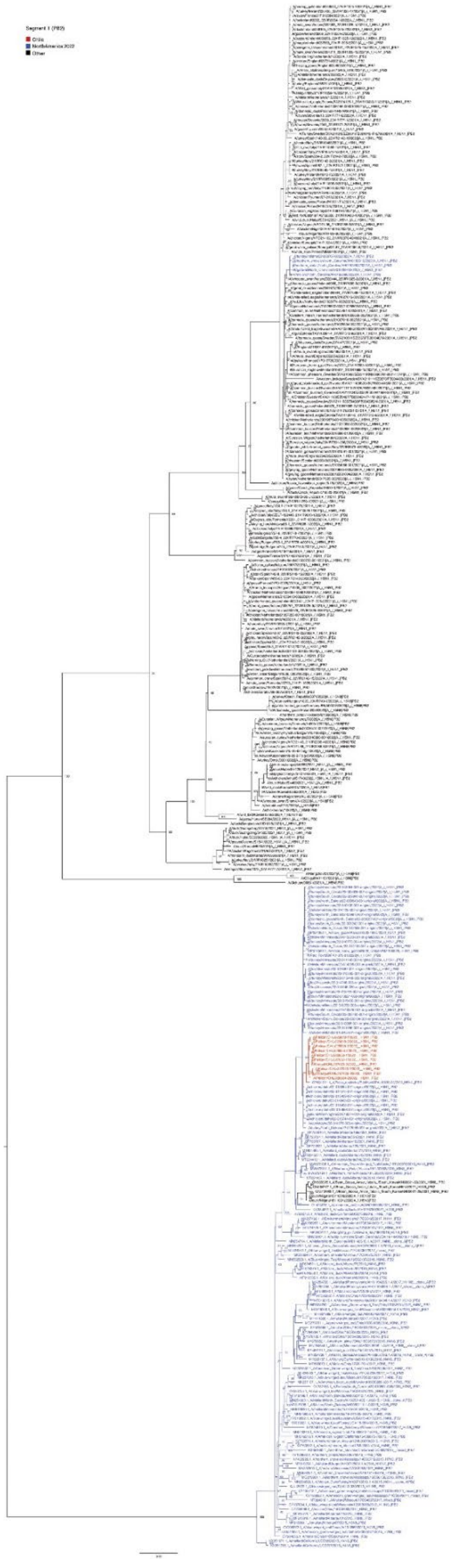

Appendix Figure 3. Maximum likelihood phylogenetic tree depicting the phylogeny of polymerase basic protein 1 (PB1) from isolates of highly pathogenic avian influenza H5N1 subtype 2.3.4.4b H5 clade virus, Chile, 2022. Scale bar indicates nucleotide substitutions per site.

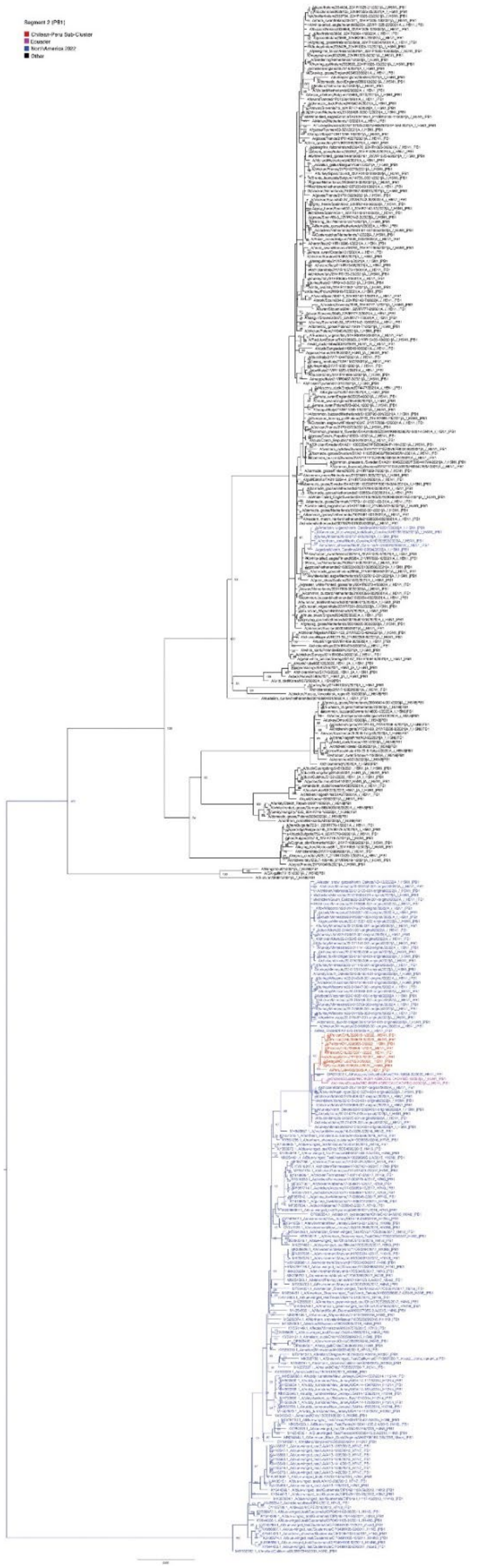

Appendix Figure 4. Maximum likelihood phylogenetic tree depicting the phylogeny of polymerase basic protein 2 (PB2) from isolates of highly pathogenic avian influenza H5N1 subtype 2.3.4.4b H5 clade virus, Chile, 2022. Scale bar indicates nucleotide substitutions per site.

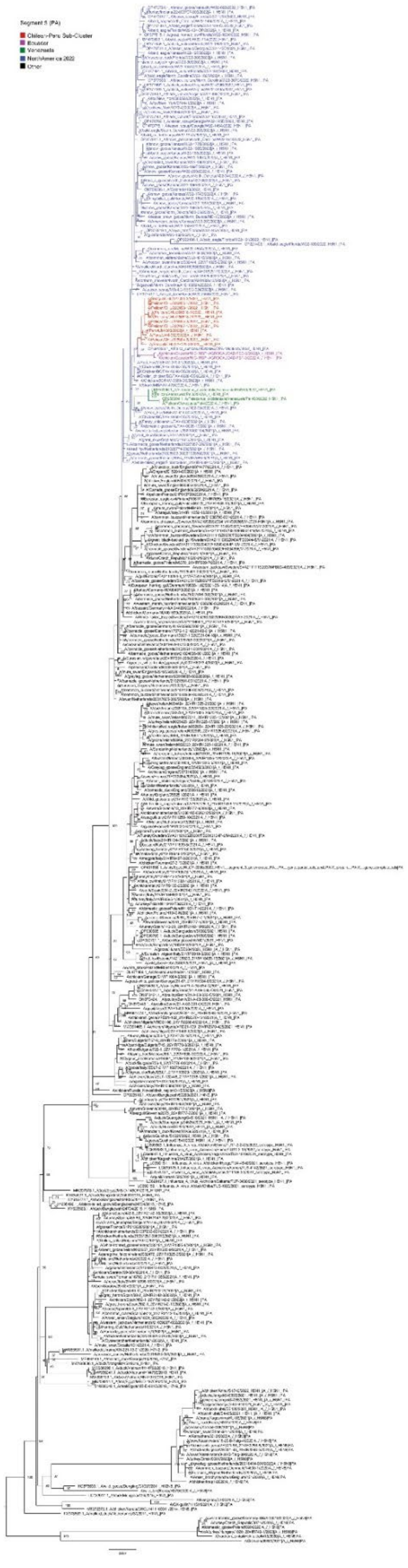

Appendix Figure 5. Maximum likelihood phylogenetic tree depicting the phylogeny of polymerase acidic (PA) segment from isolates of highly pathogenic avian influenza H5N1 subtype 2.3.4.4b H5 clade virus, Chile, 2022. Scale bar indicates nucleotide substitutions per site.

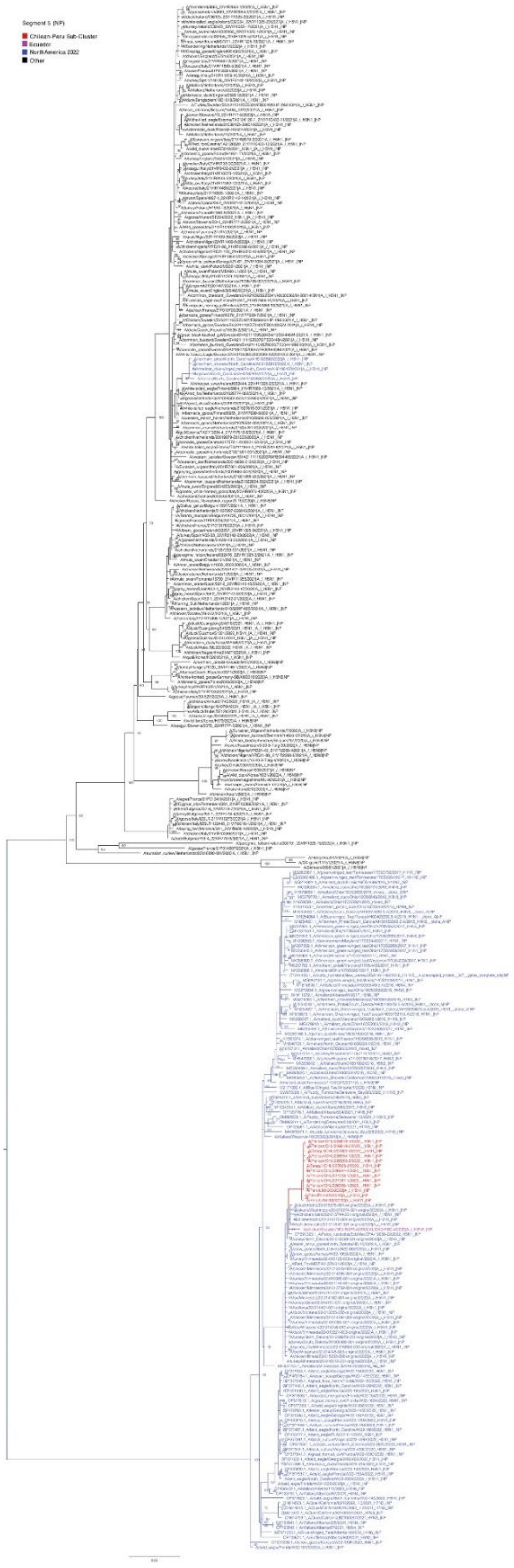

Appendix Figure 6. Maximum likelihood phylogenetic tree depicting the phylogeny of nucleoprotein (NP) segment from isolates of highly pathogenic avian influenza H5N1 subtype 2.3.4.4b H5 clade virus, Chile, 2022. Scale bar indicates nucleotide substitutions per site.

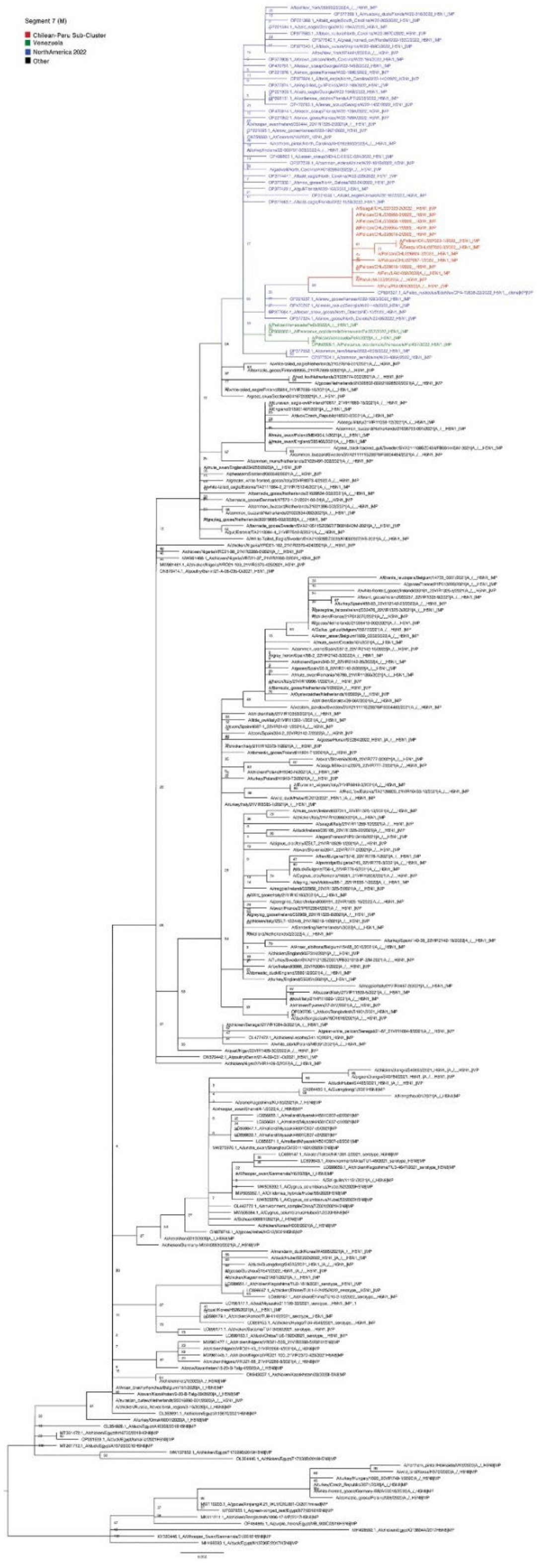

Appendix Figure 7. Maximum likelihood phylogenetic tree depicting the phylogeny of matrix (M) segment from isolates of highly pathogenic avian influenza H5N1 subtype 2.3.4.4b H5 clade virus, Chile, 2022. Scale bar indicates nucleotide substitutions per site.

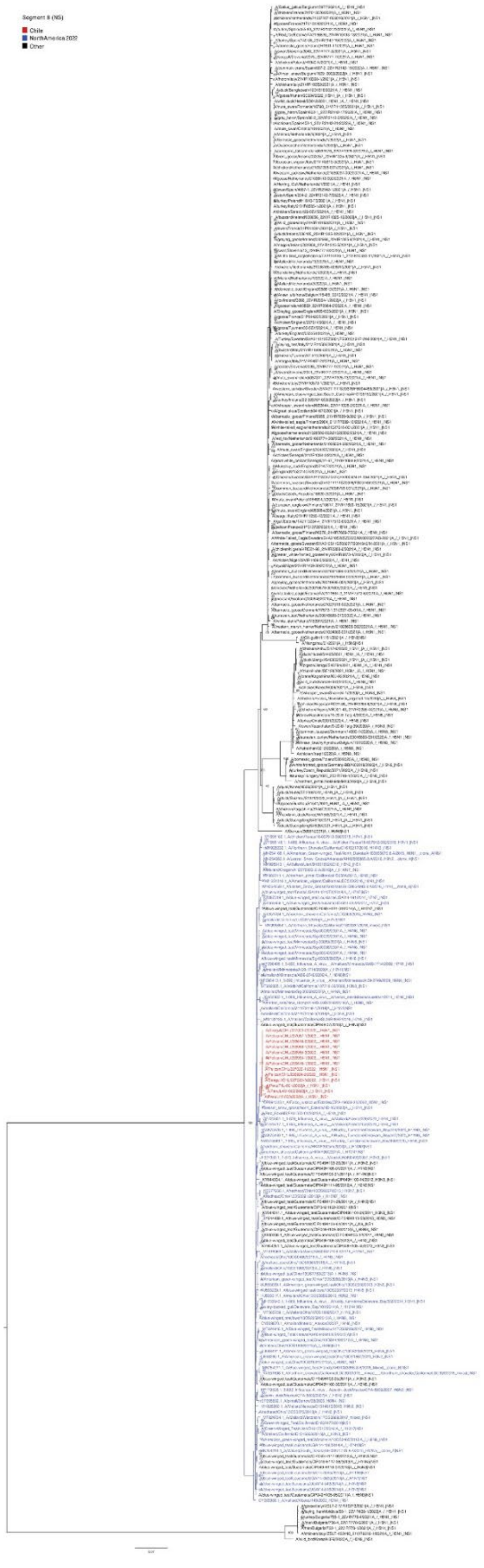

Appendix Figure 8. Maximum likelihood phylogenetic tree depicting the phylogeny of nonstructural protein (NP) from isolates of highly pathogenic avian influenza H5N1 subtype 2.3.4.4b H5 clade virus, Chile, 2022. Scale bar indicates nucleotide substitutions per site.
